# Morphogenesis and cell wall composition of trichomes and their function in response to salt in halophyte *Salsola ferganica*

**DOI:** 10.1186/s12870-022-03933-x

**Published:** 2022-11-30

**Authors:** Yanxia Liu, Yali Ma, Hanat Aray, Haiyan Lan

**Affiliations:** 1 Xinjiang Key Laboratory of Biological Resources and Genetic Engineering, College of Life Science and Technology, Xinjiang University, Urumqi, 830017 China; 2 Xinjiang Education College, Urumqi, 830043 China

**Keywords:** Cell wall composition, Gene expression, Morphogenesis, Trichome, *Salsola ferganica*

## Abstract

**Background:**

To survive harsh environmental conditions, desert plants show various adaptions, such as the evolution of trichomes, which are protective epidermal protrusions. Currently, the morphogenesis and function of trichomes in desert plants are not well understood. *Salsola ferganica* is an annual halophyte distributed in cold deserts; at the seedling stage, its rod-shaped true leaves are covered with long and thick trichomes and are affected by habitat conditions. Therefore, we evaluated the trichomes on morphogenesis and cell wall composition of *S. ferganica* compared to *Arabidopsis thaliana* and cotton, related gene expression, and preliminary function in salt accumulation of the leaves.

**Results:**

The trichomes of *S. ferganica* were initiated from the epidermal primordium, followed by two to three rounds of cell division to form a multicellular trichome, while some genes associated with them were positively involved. Cell wall composition analysis showed that different polysaccharides including heavily methyl-esterified and fully de-esterified pectins (before maturation, probably in the primary wall), xyloglucans (in the mid-early and middle stages, probably in the secondary wall), and extensin (during the whole developmental period) were detected, which were different from those found in trichomes of *Arabidopsis* and cotton. Moreover, trichome development was affected by abiotic stress, and might accumulate salt from the mesophyll cells and secrete outside.

**Conclusions:**

*S. ferganica* has multicellular, non-branched trichomes that undergo two to three rounds of cell division and are affected by abiotic stress. They have a unique cell wall composition which is different from that of *Arabidopsis* and cotton. Furthermore, several genes positively or negatively regulate trichome development. Our findings should contribute to our further understanding of the biogenesis and adaptation of plant accessory structures in desert plant species.

**Supplementary Information:**

The online version contains supplementary material available at 10.1186/s12870-022-03933-x.

## Background

Trichomes are specialised epidermal appendages found on the surface of aerial organs of most land plants, and their morphology may be a useful diagnostic characteristic of the family [1, 2]. Even on a single plant, trichomes may vary in different tissues, organs, or locations. For example, cultivated tomato (*Solanum lycopersicum*) produces several different types of trichomes on hypocotyls, stems, and leaves [3]; Certain species in the Lychnophorinae have great diversity of trichomes, including needle-, stellate-, geminate- or bladder-like forms [4]. Furthermore, trichomes have variable cell types and morphological characteristics ranging from unicellular and simple structures to multicellular [5]. For example, trichomes of *Gossypium hirsutum* and *Arabidopsis thaliana* are unicellular [6], whereas those of *S. lycopersicum* and *Withania somnifera* [7, 8] are multicellular. However, research so far in non-glandular trichomes has been mainly focused on *A. thaliana* and cotton.

Trichome development in *A. thaliana* occurs in four stages: initiation, branching, extension, and morphogenesis. Trichomes develop from protodermal cells as they undergo four cycles of endoreduplication and rapid cell enlargement [9]. Unlike *Arabidopsis*, cotton fibres differentiate from the ovule epidermis and develop in four stages: fibre initiation, elongation, secondary cell wall (SCW) biosynthesis, and maturation [10]. Non-glandular trichomes, such as those observed in *A. thaliana* and *G. hirsutum*, have typically been used as model systems for studying cell differentiation [11]. Trichome growth and development are controlled by genetic and cellular programs that respond to many endogenous and environmental signals [2]. Correspondingly, trichome development is complex and involves a series of genes that regulate trichome spacing, density, and morphology [11]. Expression pattern analysis of these genes may aid in understanding the molecular mechanisms that control cell fate, differentiation, and function in plants [6, 11].

Plant growth and morphogenesis are strongly dependent on the dynamic structure of the cell wall [10, 12]. The construction of the unique structures of trichomes requires a highly diverse polysaccharide assemblage [13, 14]. Knowledge of the structure and content of each cell wall polymer is a prerequisite for understanding their functions during plant development and adaptation [15]. Pectins control cell wall flexibility and are thus crucial for cell proliferation and plant growth [16], while xyloglucans (XGs) are essential for conveying biomechanical stability to walls [17]. Extensin participates in physiological activities and plays a vital role in plant development and stress tolerance [18]. The cell wall of root hairs in *Arabidopsis* consists of arabinogalactan proteins (AGPs) and XGs [19]. In cotton, the epidermal layer of fibres exhibits a higher rhamnogalacturonan I (RGI)-AGP ratio than other ovular tissues. To date, research on trichome cell walls has been focused on a few plant species, and information regarding the trichomes of non-model plants is limited.

Trichomes perform multiple functions, such as defending against insects and pathogens, decreasing water loss, and preventing UV damage [20]. Moreover, trichomes play an important role in maintaining plant function after exposure to environmental stress [21]. They can respond to salinity by changing their density [22] and even influence cell enlargement [23]. Furthermore, trichomes can also excrete excess Na^+^ under salt stress, contributing to plant survival in saline environments [24]. This is an indication that some species might develop different functional and structural mechanisms based on tolerance in a specific environment [25].

*S. ferganica* is an annual desert halophyte belonging to the Chenopodiaceae family. In a previous study, we found that, at the seedling stage, the rod-shaped true leaves of *S. ferganica* were covered with long, thick, and white trichomes, which became shorter and thinner at later developmental stages. Furthermore, the length and density of trichomes on the abaxial surface of true leaves were significantly higher than those on the adaxial surface and were affected by habitat conditions [26].

In the present study, we aimed to investigate (1) the morphogenesis of trichomes in *S. ferganica*, (2) trichome development and associated changes in cell wall polysaccharides and trichome-related gene expression during this process, and (3) function of trichomes in response to salt.

## Results

### Morphology of the trichomes during seedling development in *S. ferganica*

**Fig. 1:**
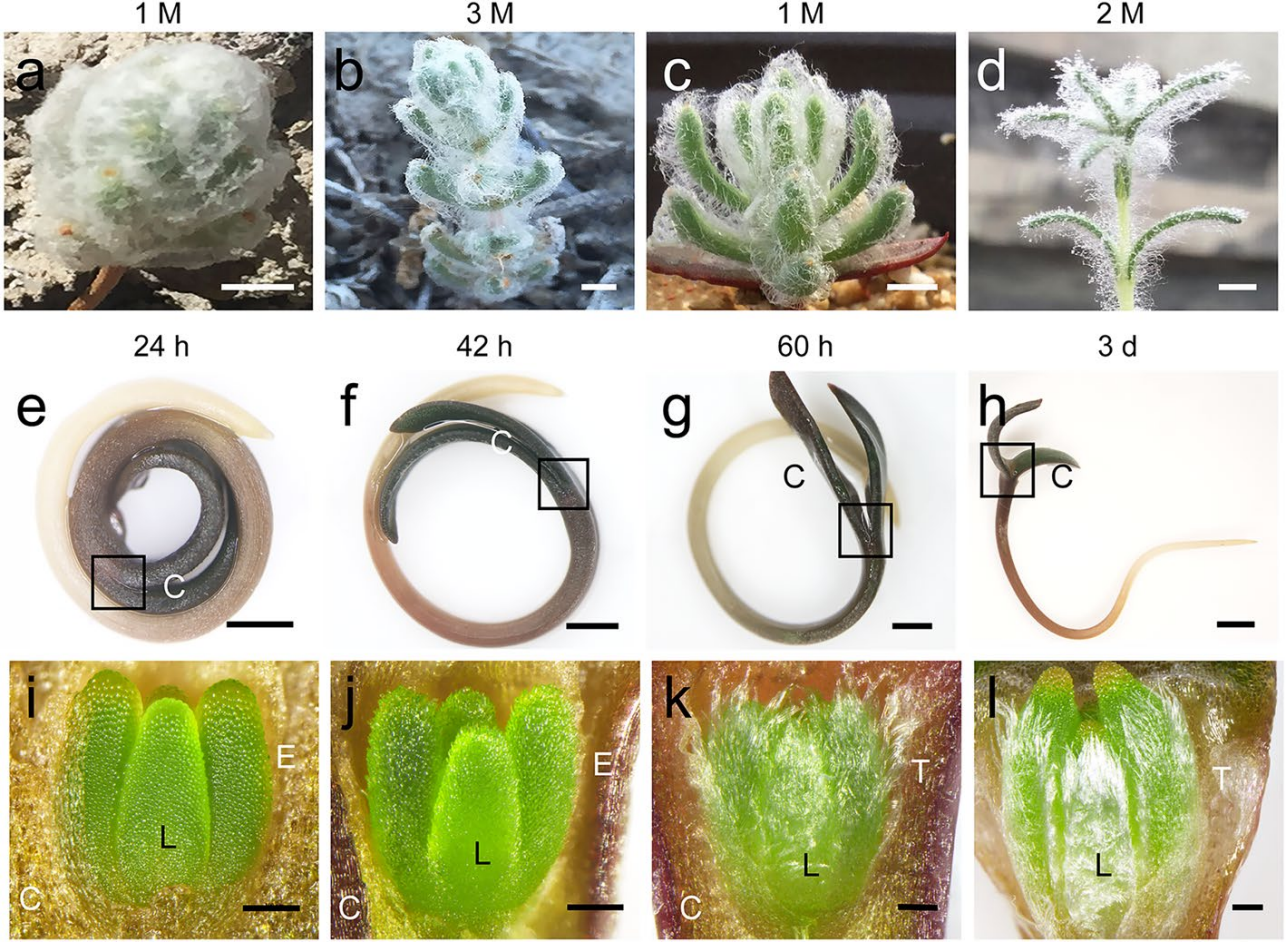
Morphology of the trichomes in seedling development in *S. ferganica*. **a–b** Trichomes on a seedling grown in the natural habitat. **c–d** Trichomes on a seedling grown indoors. **e–h** Germination process (at 24 h, 36 h, 60 h, and 3 d of seed germination). **i–l** The true leaves and trichomes at the growth point between two cotyledons under a stereomicroscope (corresponding to that of **e–h** in the boxes). Scale bar: **a–d**, 500 μm; **e–g**, 1000 μm; **h**, 2000 μm; **i–k**, 150 μm; **l**, 250 μm. C: cotyledon; L: leaf; E: epidermal cell; T: mature trichomes

*S. ferganica* possesses a unique feature—the early seedling is covered with long, thick, and white trichomes, while the cotyledon is naked without any hairs throughout the development of the leaf (Fig. S1). Furthermore, seedlings in the natural habitat presented a higher density of trichomes than those grown indoors (Fig. 1a–d). From 24 to 36 h after germination, granular protrusions were visible on the newly developed true leaves (Fig. 1e, f, i, j). When germination occurred at 60 h, trichomes on the abaxial leaf surface elongated (Fig. 1g, k) for up to 3 d and became thicker till 3 d (Fig. 1h, l). However, the true leaf was still not visible to the naked eye at the growth point of the fully expanded cotyledons (Fig. 1h, l). These data suggest that trichome formation initiates in the early stages of true leaf development during seed germination.

### The microstructure of the trichomes and their distribution on the leaf in *S. ferganica*

**Fig. 2:**
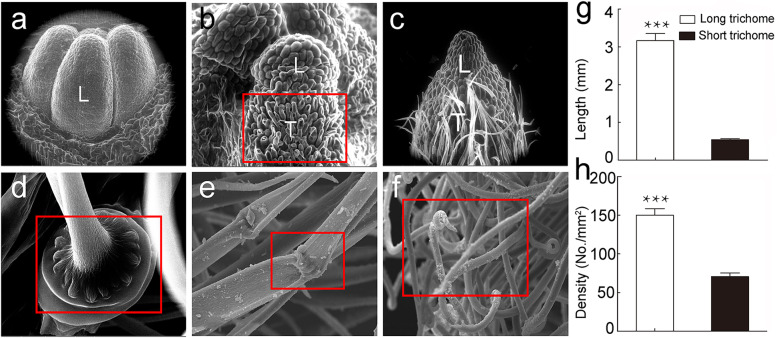
Morphological characteristics and some statistical indexes of trichomes on the leaves of *S. ferganica*. **a**–**c** ESEM images showing trichome development from initiation to maturation. **d**–**f** SEM images showing the microstructure of the trichome at the base, internode, and tip. **g**, **h** Statistical length and density of trichomes. ***: represents the significant difference (*P* < 0.05 or 0.01) between long and short trichomes. Values are means ± SD of at least nine replicates. L: leaf; T: trichomes

To further characterise the micromorphology of the trichomes of *S. ferganica*, the biogenesis and microstructure of trichomes were visualised under the ESEM and SEM (Fig. 2a–f). Trichome initiation is intimately coupled with leaf development. Initially, we could only see four leaf primordia at the apical growth point without any protrusion of trichome cells on the leaf surface (Fig. 2a). With the development of the leaf, the primary trichome cell bulged and elongated (Fig. 2b), and finally, the trichomes became visible at the base and middle parts of the leaf (Fig. 2c). At maturity, trichomes were classified into two types: long (abaxial surface) and short (adaxial surface of leaf) trichomes. A single trichome consisted of 1–2 expanded basal cell, upper long-stalk cells, and a coiled tip, with 2–3 internodes in between (Fig. 2d–f). The leaves of *S. ferganica* were divided into abaxial (with long trichomes) and adaxial (short trichomes) surfaces. In 6-week-old *S. ferganica*, the length and density of long trichomes were approximately three times greater than those of short trichomes (Fig. 2g–h).

### Morphogenesis of the trichomes in *S. ferganica* and proposed model

**Fig. 3:**
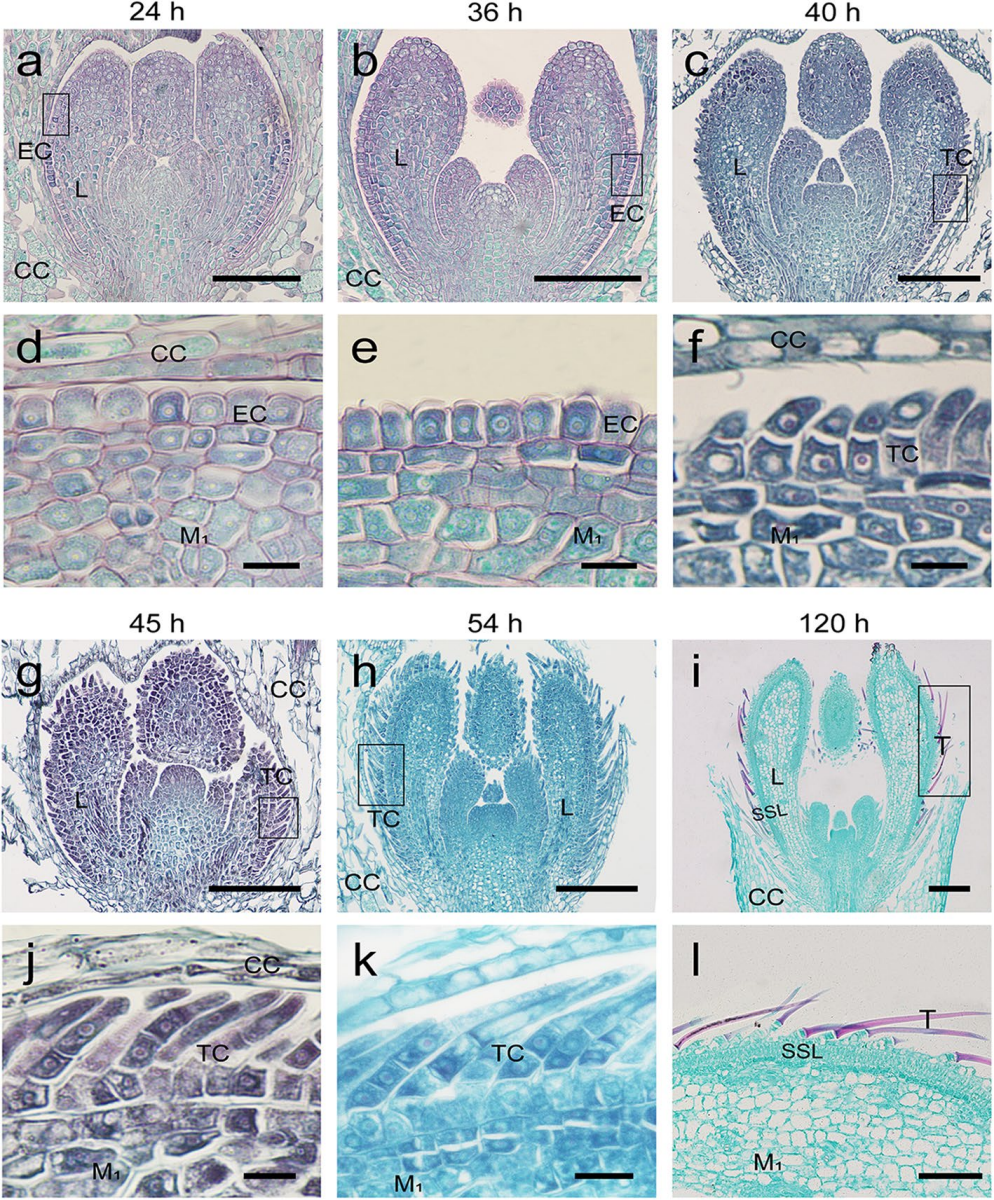
Cytological changes in trichomes during leaf development in *S. ferganica*. **a**–**c**, **g–i** Longitudinal sections of the true leaf primordia between two cotyledons. **d–f**, **j–l** Local enlarged images showing the trichome cell development (corresponding to images in boxes marked in **a–c** and **g–i**, respectively). **a**, **d** Germination for 24 h; **b**, **e** 36 h; **c, f** 40 h; **g**, **j** 45 h; **h**, **k** 54 h; **i**, **l** 120 h. Scale bar: **a**–**c**, **g**, **h**, 100 μm; **d–f**, **j**, 10 μm; **i**, 1000 μm; **k**, 20 μm; **l**, 500 μm. EC: epidermal cell; CC: cotyledon cell; M1: mesophyll; TC: trichome cell; L: leaf; T: mature trichomes; SSL: salt-storage layer

Cytological processes during trichome development were observed. Within approximately 24 h of germination, the epidermal cells on the abaxial surface of the newly developed true leaves showed no significant difference compared to mesophyll cells (Fig. 3a, d). During leaf development, some epidermal cells elongated longitudinally and could be distinguished from other epidermal cells, which were the initial trichome cells (Fig. 3b, e). Subsequently, the well-developed initial cell divided to form a basal cell and an apical cell (Fig. 3c, f). After two or three divisions, the multicellular trichomes began to differentiate (Fig. 3g, h, j, k). The top cell extended rapidly and became conically shaped, and was longer than the cells in the lower part (Fig. 3i, l). Our results indicate that the trichomes of *S. ferganica* are multicellular, and their formation initiates after true leaf primordium emergence.

We compared the developmental process of trichomes in *S. ferganica* using *G. hirsutum* and *A. thaliana* as controls (Fig. S2) because trichome development has been well documented for these species. Paraffin sections showed that, in *G. hirsutum*, some outer-integument cells of the ovule started to initiate at 1 d before anthesis and were significantly elongated at 3 d post-anthesis (DPA). With the ovule development, the fibre grew rapidly at 15 DPA. The fibre might be absent in living substances, and the testa became too hard to be sectioned intactly. The cotton seed trichomes are single-cellular and elongated without any division or branching. In *A. thaliana*, trichome development comprises epidermal cell expansion (germination for 3 d), branching (5–6 d), extension (after 6 d), and maturation (after approximately 10 d), finally forming a three-branched single-cellular trichome.

**Fig. 4:**
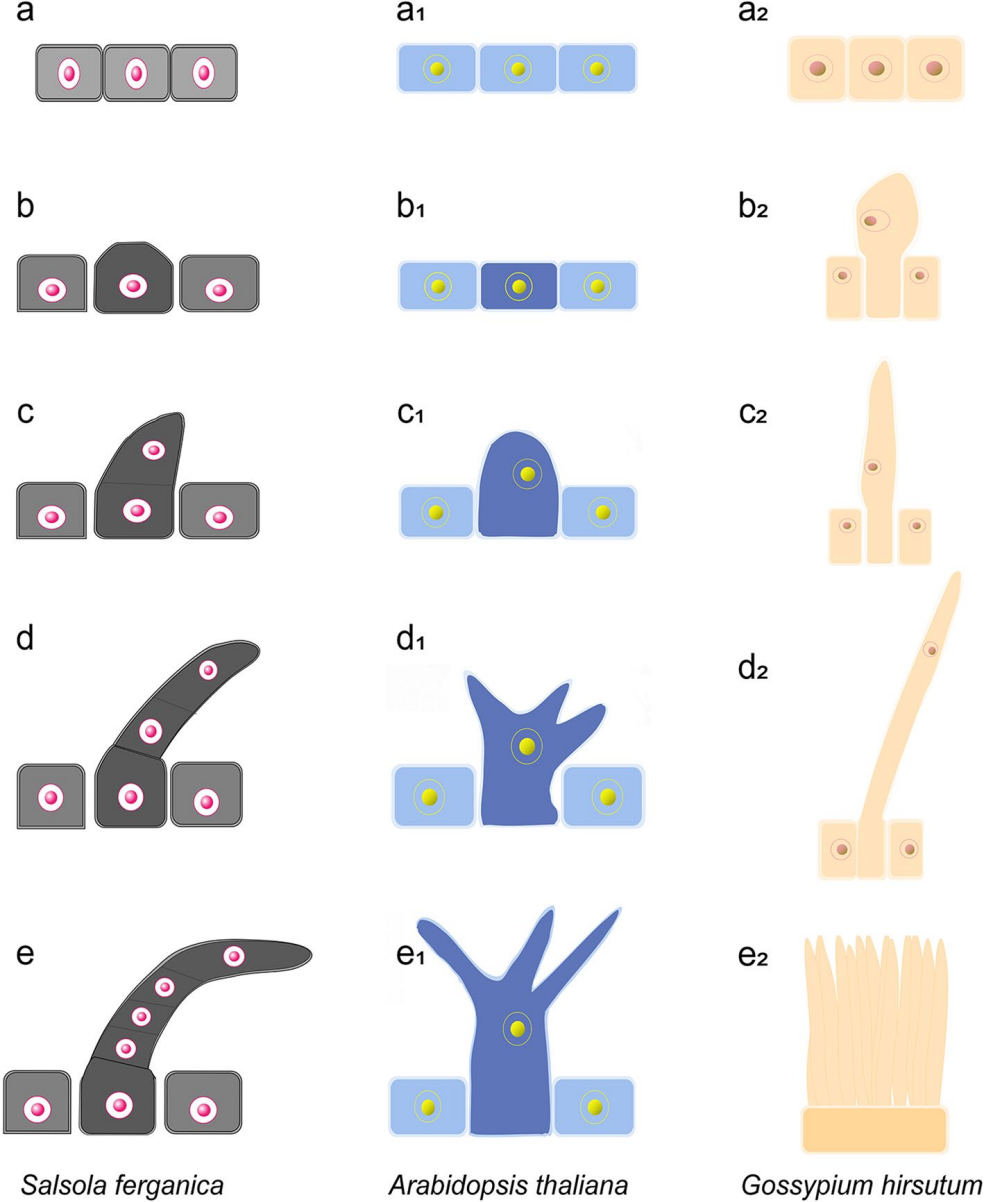
A proposed model for the biogenesis of trichomes in *S. ferganica*. **a**–**e** *S. ferganica*. **a**_**1**_–**e**_**1**_ *G. hirsutum*. **a**_**2**_–**e**_**2**_ *A. thaliana*. **a**, **a**_**1**_, **a**_**2**_: showing epidermal cells

Based on our observation of paraffin sections, we proposed a model of trichome biogenesis in *S. ferganica* using *Arabidopsis* and cotton seed trichomes as controls (Fig. 4). In *S. ferganica*, the initiation of trichome cells began at approximately 24 h after germination (Fig. 4b), followed by 2–3 cell divisions, differentiation, and development, finally resulting in the formation of a multicellular, unbranched trichome (Fig. 4c–e). In *Arabidopsis* and *G. hirsutum*, early trichome cells also experienced the protrusion of epidermal cells; however, instead of undergoing cell division, they branched or elongated, respectively (Fig. 4a_1_–e_1_, a_2_–e_2_).

### Dynamic changes in polysaccharides in the trichome cell wall in *S. ferganica*

When labelled with JIM5, which recognises low methyl-esterified homogalacturonan (LMHG), *S. ferganica* trichomes presented no obviously labelling signal, which is significantly different from those of *G. hirsutum* and *A. thaliana* (Fig. S3). In *G. hirsutum*, JIM5 signal was gradually enhanced in the fibres after anthesis (Fig. S3); in *A. thaliana*, JIM5 signal was strongly detected in trichomes rather than the other epidermal cells and mesophyll cells in development (Fig. S3). These data indicate that LMHG has different distribution patterns among the three species.

JIM7 labelling, which recognises heavily methyl-esterified homogalacturonan (HMHG), of *S. ferganica* trichomes revealed a similar pattern to those of *G. hirsutum* and *A. thaliana*, which showed strong JIM7 signals during development (Fig. S4), indicating the presence of large amounts of HMHG in the trichome cell walls.

The antibody CCRC-M38 can recognise the fully de-esterified homogalacturonan (FDEHG). The trichomes of *S. ferganica* were relatively less labelled during development, and the colour was mainly present on the cell wall between the two cells and inside the trichome cell (Fig. S5).  For *G*. *hirsutum* and *A. thaliana*, trichomes were moderately labelled at each stage. Furthermore, FDEHGs in the trichomes appeared to be enhanced in the mature cell wall in all the three species.

The antibody CCRC-M7 recognises RGI and AGP. CCRC-M7 treatment of the trichomes of *S. ferganica* revealed no labelling during development, which is different from that of *G. hirsutum* (Fig. S6). In *G. hirsutum*, moderate labelling was present in the early fibres (before 2 DPA), but it gradually reduced with development (after 6 DPA). In contrast, the trichomes of *A. thaliana* were strongly labelled in the cell wall. Our results suggest that RGI and AGP are differentially distributed in the trichomes of the three species.

When labelled with CCRC-M1, which recognises XGs, trichomes of *S. ferganica* presented a darker colour on the cell wall compared with the trichomes of cotton and *Arabidopsis* (Fig. S7). With trichome maturation, the significantly elongated top cell, but not the cells in the lower segment, did not show labelling in *S. ferganica*; cotton and *Arabidopsis* trichomes were weakly labelled during the development except for the very early stage in *Arabidopsis* (Fig. S7). The results suggest that XGs are gradually reduced with trichome development.

**Fig. 5:**
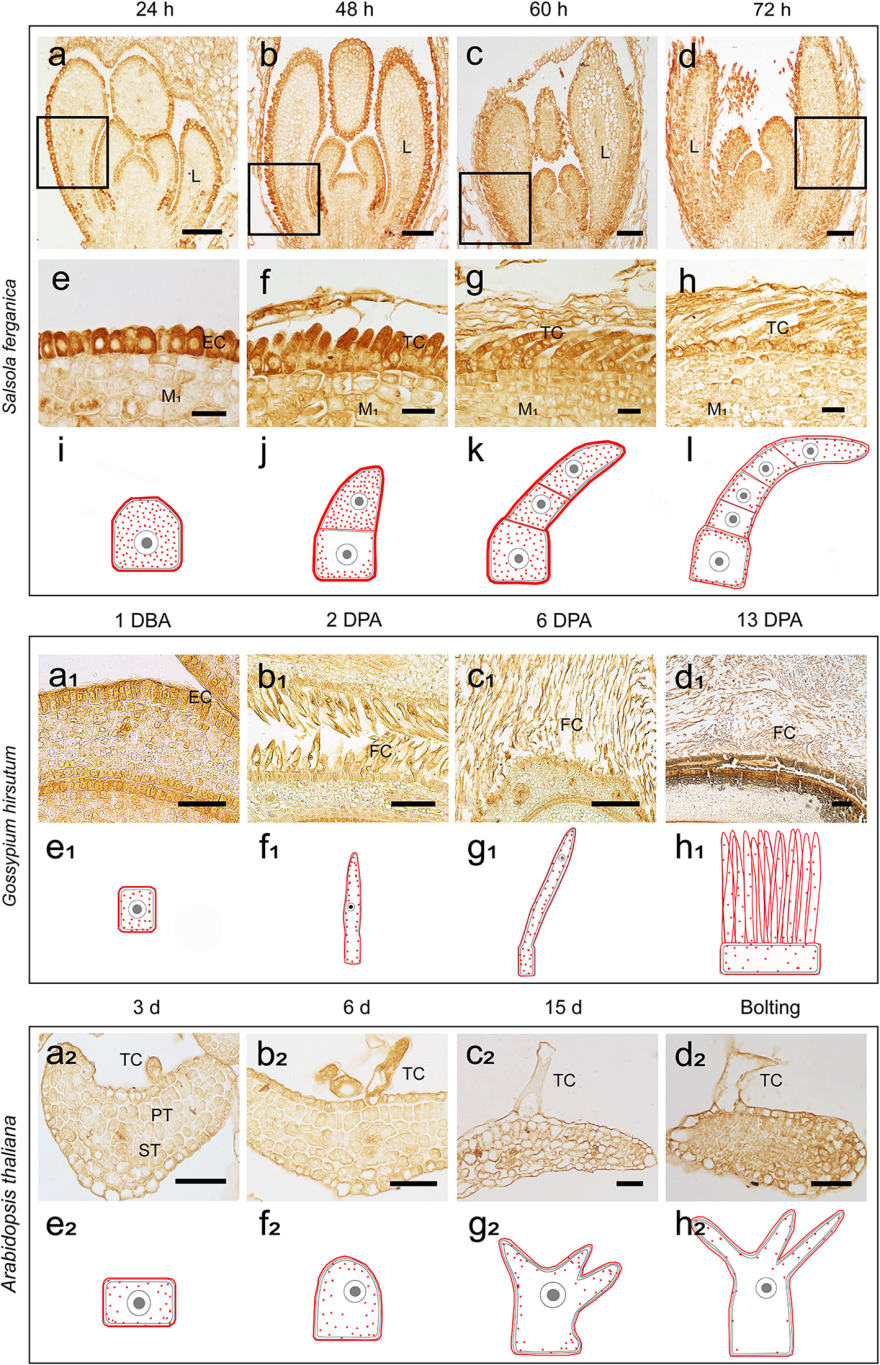
Localisation of extensin (recognised by LM1) in the trichomes of *S. ferganica*. **a**–**l** Extensin localisation in trichomes of *S. ferganica* at different developmental stages. **e**–**h** Local enlarged views corresponding to that of **a–d** in the boxes. **i**–**l** Hand-drawn diagram of trichomes in the developmental process showing extensin localisation. **a**, **e**, **i** Germination for 24 ± 12 h; **b**, **f**, **j** 48 ± 12 h; **c**, **g**, **k** 60 ± 12 h; **d**, **h**, **l** 72 ± 12 h. **a**_**1**_-**h**_**1**_ Extensin localisation in fibres of *G. hirsutum*. **e**_**1**_–**h**_**1**_ Hand-drawn diagram of fibres in the developmental process showing extensin localisation. **a**_**1**_, **e**_**1**_ 1 DBA; **b**_**1**_, **f**_**1**_ 2 DPA; **c**_**1**_, **g**_**1**_ 6 DPA; **d**_**1**_, **h**_**1**_ 13 DPA. **a**_**2**_–**h**_**2**_ Extensin localisation in trichomes of *A. thaliana*. **e**_**2**_–**h**_**2**_ Hand-drawn diagram of trichomes in the developmental process showing extensin localisation. **a**_**2**_, **e**_**2**_ Germination for 3 d; **b**_**2**_, **f**_**2**_ 6 d; **c**_**2**_, **g**_**2**_ 15 d; **d**_**2**_, **h**_**2**_ Bolting. Scale bar: **a**–**d**, 100 μm; **e**–**h**, 25 μm; **a**_**1**_, **b**_**1**_, is 50 μm; **c**_**1**_, **d**_**1**_, 200 μm; **a**_**2**_–**d**_**2**_, 40 μm. h: hour; DBA: days before anthesis; DPA: days post anthesis; d: day; L: leaf; EC: epidermal cell; TC: trichome cell; M1: mesophyll; FC: fibre cell; PT: palisade tissue; ST: spongy tissue

The LM1 labelling of *S. ferganica* trichomes was distinctly and strongly detected in the trichome cell and cell wall, especially in the top cell at the early stage, which is significantly different from that of cotton and *Arabidopsis* (Fig. 5). LM1 was moderately labelled in the trichomes of *G. hirsutum* and *Arabidopsis* at an early stage and was significantly reduced in the later stages (Fig. 5). These results indicate that extensin plays an important role in the biogenesis and extension of trichome cells in *S. ferganica*.

Immunohistochemical analysis revealed significant differences in cell wall components of *S. ferganica*, *Arabidopsis*, and cotton (Table 1, Table S2). Our results showed that highly esterified HG (recognised by JIM7) and de-esterified HG (recognised by CCRC-M38) were commonly distributed in the trichomes of *S. ferganica* and decreased at the later stage. Meanwhile, extensin (recognised by LM1) was strongly labelled in *S. ferganica* compared to that in *Arabidopsis* and cotton. Partially methyl-esterified HG (recognised by JIM5) and RGΙ (recognised by CCRC-M7) showed weaker labelling, while XG (recognised by CCRC-M1) was abundant in the early stage but decreased in the later stage of trichomes development in *S. ferganica*. The cell wall components of the trichomes in *Arabidopsis* and cotton were similar, showing the presence of low methyl-esterified, heavily methyl-esterified, or fully de-esterified HGs; the contents of RGI/AGP and extensin were relatively higher, and XG was also detected in both trichomes.

**Table 1:** Antibodies employed in the present study and their labelling intensity on trichomes of *S. ferganica*

Antibody	Recognition of antigen	*S*. *ferganica*	*G. hirsutum*	*A. thaliana*
JIM5	Pectin (low methyl-esterified)	–	+	++
JIM7	Pectin (heavily methyl-esterified)	++	++	+
CCRC-M38	Pectin (fully de-esterified)	+	+	+
CCRC-M7	RGI and AGP	–	++	++
CCRC-M1	Xyloglucan	++	+	+
LM1	Extensin	+++	++	++

‘+’ represents the labelling intensity of the antibody (+, labelled; ++, relatively stronger; +++, strong); ‘-’ represents no labelling or much weak labelling. Negative control (see Fig. S8)

### Expression patterns of trichome-related genes in *S. ferganica*

**Fig. 6:**
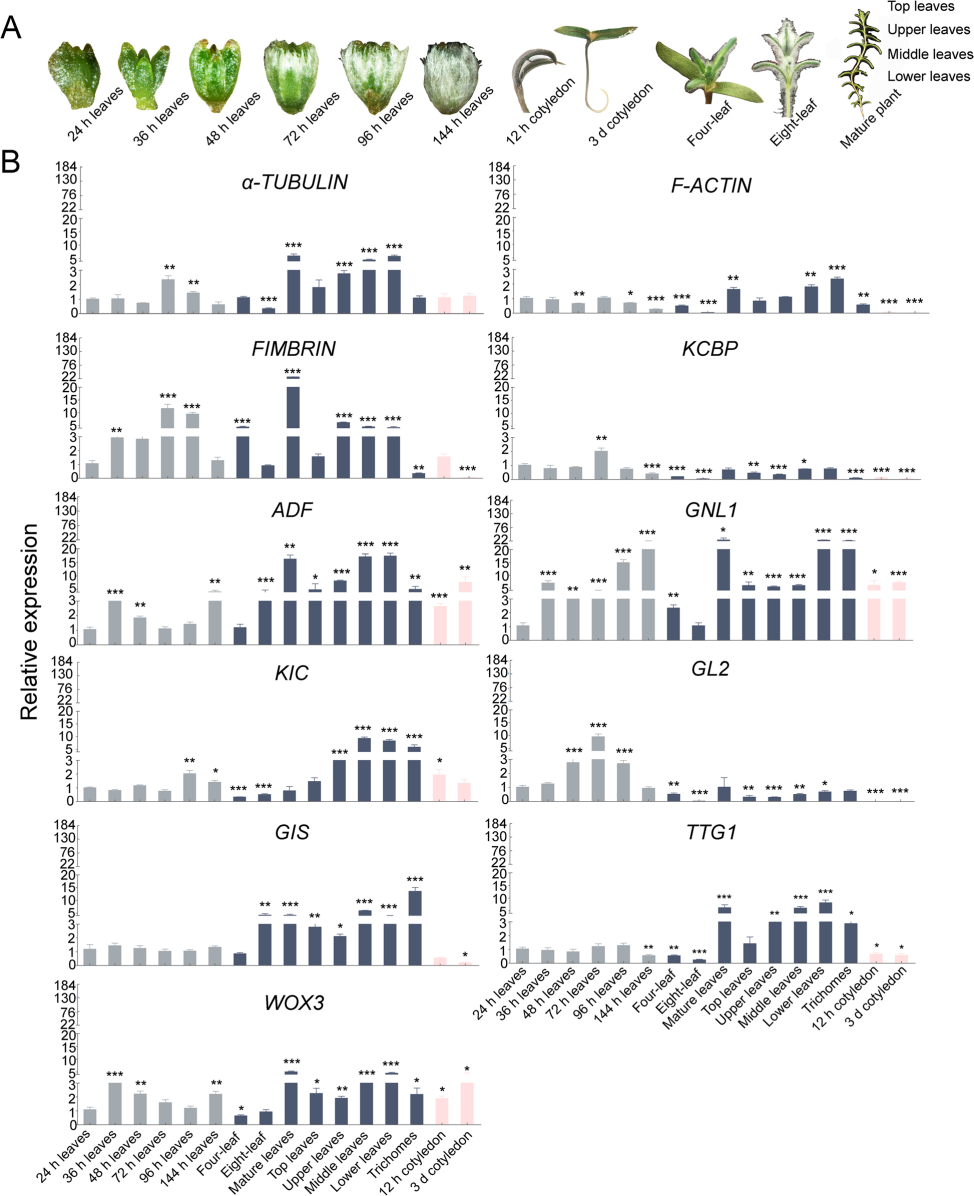
Expression patterns of trichome-related genes in *S. ferganica*. **a** Morphology of *S. ferganica* at different stages. **b** *α-TUBULIN*, *F-ACTIN*, *FIMBRIN*, *KCBP*, *ADF*: microfilament and microtubule regulated genes; *GNL1*: vesicle transport-related gene; *KIC*: calcium ion transport-related gene; *GL2*, *GIS*, *TTG1*, *WOX3*: trichome development-related genes. Grey columns represent early stages of trichome development; dark blue columns represent later stages of trichome development; pink columns represent cotyledon stages. 24 h, 36 h, 48 h, 72 h, 96 h, and 144 h leaves: represent leaves sampled from seedlings after germination for 24 h, 36 h, 48 h, 72 h, 96 h, and 144 h, respectively; Four-leaf, eight-leaf: sampling leaves from seedlings with four or eight leaves; mature leaves: sampling leaves from the adult plant; top, upper, middle, lower leaves: sampling leaves from the top, upper, middle, and lower part of the plant; trichome: sampling trichomes from the senescent leaves;12 h, 3 d cotyledon: sampling cotyledons from seedlings after germination for 12 h, 3 d. *,**,***: represent significant differences (*P* < 0.05, 0.01, 0.001, respectively) between the expression level in leaves at 24 h germination and at other time points. Values are means ± SD of six replicates (three biological replicates with two technical repeats of each)

Based on the morphogenesis of trichomes in *S. ferganica*, we further analysed the expression patterns of some trichome-related genes during development (Fig. 6). Results showed that microtubule-related genes—*α-TUBULIN*, *FIMBRIN* (actin-bundling protein), and *KCBP* (kinesin-like calmodulin binding protein)—were upregulated at the early developmental stage with the highest expression level on the third day, and the first two genes presented relatively higher expression levels during leaf maturation stages; all three genes were down-regulated in the trichomes of senescent leaves and cotyledons (Fig. 6). Microfilaments may regulate the elongation of trichome cells [27]. In the present study, the expression level of *F-ACTIN* increased at mature leaf stages and was much lower in cotyledons and trichomes of senescent leaves, whereas the transcripts of actin depolymerising factor (*ADF*) gene were accumulated significantly at early and middle developmental stages. Golgi stacks may play a role in trichome morphogenesis; according to previous studies, expression of vesicle-associated genes, such as GNOM-like 1 (*GNL1*), has a significant impact on Golgi traffic [28, 29]. The results of this study showed that it was actively expressed in the early and later developmental stages of trichomes and in cotyledons. Moreover, kinesin-interacting Ca^2+^-binding protein (*KIC*) has been reported as a negative regulator of trichomes [30], which was relatively less expressed at the early stage of trichome development, but significantly expressed at the leaf maturation stage. The genes of GLABRA2 (*GL2*) [31], glabrous inflorescence stems (*GIS*) [32], transparent testa GLABRA 1(*TTG1*) [33], and wuschel-like homeobox 3 (*WOX3*) [34] are directly related to trichome development, among which *GL2* was upregulated at the early stage of trichome biogenesis with the highest level on the third day, whereas it was downregulated in the later stage; it showed lower expression level in cotyledons. The genes of *GIS* and *TTG1* were relatively less expressed at the early stages of trichome development but had relatively higher expression at the mature leaf stage. Further, *WOX3* was upregulated in the early and later stages and in the cotyledons. These results suggest that *GL2* and *KIC* may regulate the biogenesis of trichomes and that other genes may play a role in trichome development (Fig. 6).

### Effects of salt and drought stress on trichome development in *S. ferganica*

**Fig. 7:**
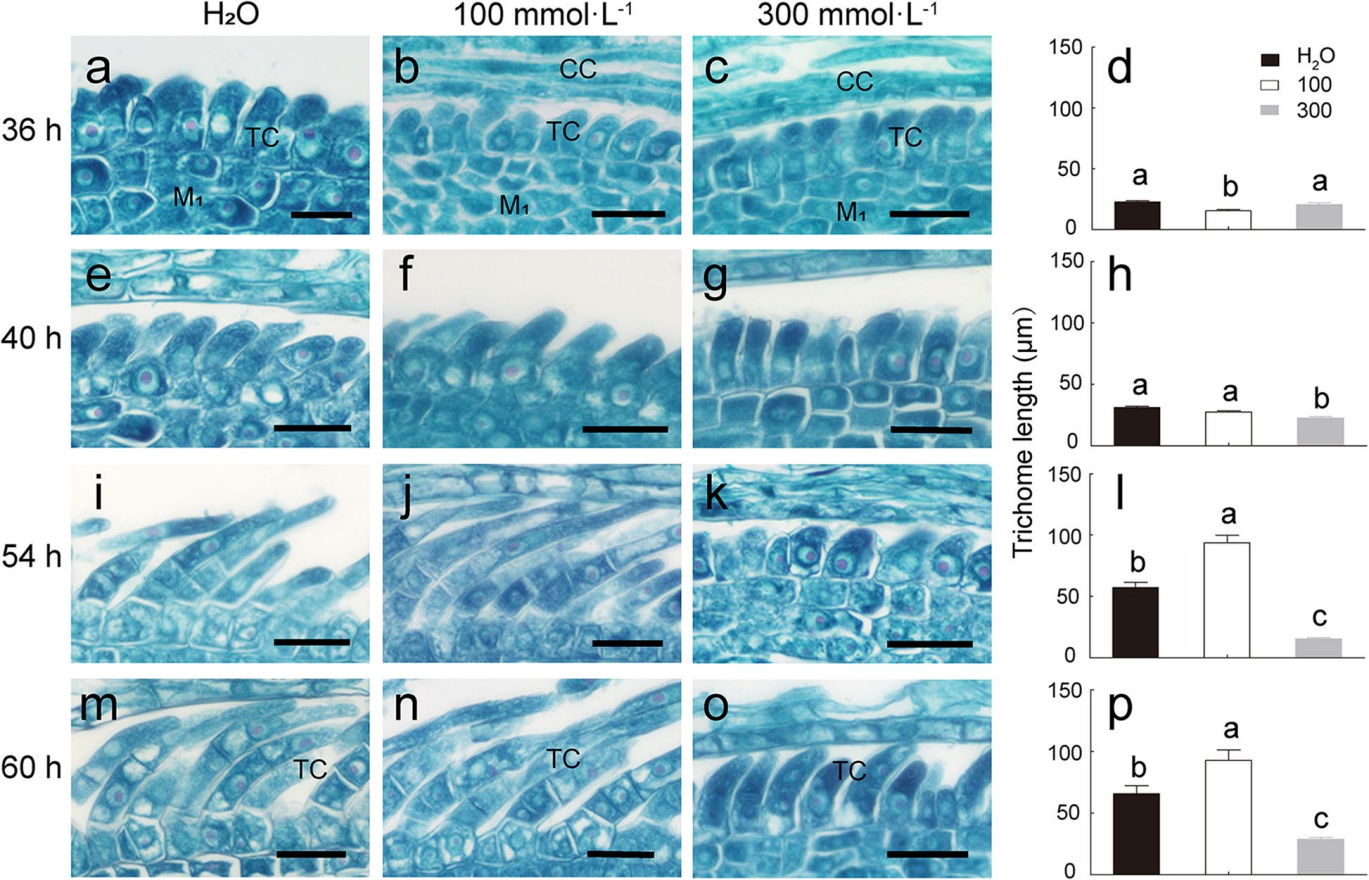
Effect of salt stress on trichome development in *S. ferganica.* **a**–**c**, **e**–**g**, **i**–**k**, **m**–**o** Cytological changes in the trichome cells during true leaf development under NaCl treatment. **d**, **h**, **l**, **p** Trichome length measurements corresponding to **a**–**c**, **e**–**g**, **i**–**k**, and **m**–**o**, respectively. **a**–**d** Germination for 36 h; **e**–**h** 40 h; **i**–**l** 54 h; **m**–**p** 60 h. **a**, **e**, **i**, **m** H_2_O; **b**, **f**, **j**, **n** 100 mmol·L^− 1^ NaCl; **c**, **g**, **k**, **o** 300 mmol·L^− 1^ NaCl. Scale bar: **a**–**c**, **e**–**g**, **i**–**k**, **m**–**o**, 25 μm. Different lowercase letters above columns indicate significant differences (*P* < 0.05 or 0.01) among different NaCl concentrations at the same developmental time. Values are means ± SD of at least 10 replicates. CC: cotyledon cell; TC: trichome cell; M1: mesophyll

Salt stress has a significant effect on trichome development, which was evaluated by observing cell structure and length of trichomes. Trichome cells grew quicker in H_2_O in the initial stage (36–40 h) of germination (Fig. 7a–h). However, in subsequent development and differentiation, which presented more advantageous under a lower NaCl concentration (100 mmol·L^− 1^) compared with the development observed in H_2_O and at a higher NaCl concentration (300 mmol·L^− 1^) (Fig. 7i–p). These results suggest that salt is not necessary to affect the trichome biogenesis; in fact, a lower NaCl concentration promotes growth, while a higher NaCl concentration inhibits the development and differentiation of the trichomes.

We chose three concentrations (0, 10, and 20% of PEG 6000) for the drought experiment based on information available in the literature [35]. The morphological observations revealed that a 20% PEG significantly inhibited plant growth. As a result, 10% PEG was employed to investigate the effect of drought stress on trichome development (Fig. S9).

Our results showed that 10% PEG did not affect trichome growth during germination at early stage (24–45 h) (Fig. S10a–f). However, significant inhibition was observed at 62 h compared with that under H_2_O. The trichomes treated with H_2_O were three times longer than those treated with 10% PEG (Fig. S10g–i). Our results suggest that even mild drought stress may inhibit trichome development in *S. ferganica*.

### Effect of salt accumulation in trichomes of *S. ferganica*

**Fig. 8:**
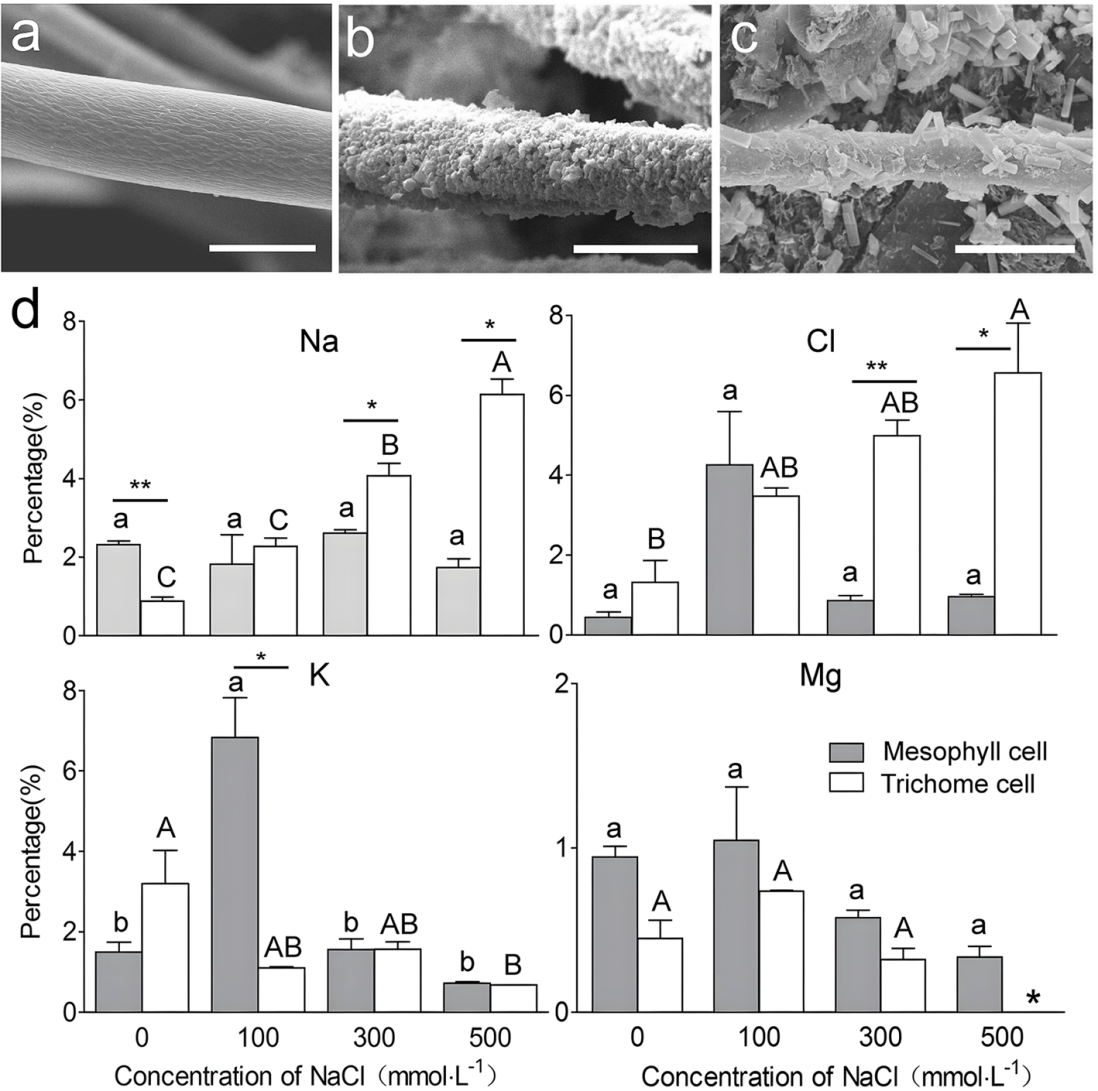
The content of different ions in trichomes of *S. ferganica* under salt stress. **a** Control (without salt treatment). **b**–**c** Microstructure of trichome surface after salt treatment (500 mmol·L^− 1^). **d** Contents of Na^+^, Cl^−^, K^+^, and Mg^2+^ in leaf mesophyll cells and trichomes. Scale bar in **a**, **b**, **c** is 20 μm. Lowercase letters indicate significant differences (*P* < 0.05) among different NaCl concentrations in mesophyll cells; uppercase letters indicate significant differences (*P* < 0.05) among different NaCl concentrations in trichomes. *,**: represents significant differences (*P* < 0.05 or 0.01) between mesophyll cells and trichomes at the same NaCl concentration. Values are means ± SD of at least three replicates

Under SEM, compared with the control without salt treatment (Fig. 8a), particles and crystal-like substances were observed on the trichome surface after 500 mmol·L^− 1^ NaCl treatment (Fig. 8b, c) . Measurement of ions showed that Na^+^ and Cl^−^ levels in trichomes were significantly higher than those in mesophyll cells at higher NaCl concentrations (300 and 500 mmol·L^− 1^); the K^+^ and Mg^2+^ levels in trichome cells were lower than these in mesophyll cells under salt stress, and the K^+^ levels in trichome and mesophyll cells did not change significantly upon treatment with salt concentrations greater than 300 mmol·L^− 1^ (Fig. 8d).

## Discussion

The plants growing in deserts have evolved various strategies to adjust to the harsh environmental conditions [36]. Trichomes are natural barriers between plants and the external environments, protecting plants against environmental stress, and can be a selective strategy for species reproduction and transmission [20]. However, only the model plants have mainly been studied on the trichomes, and relatively less research has involved non-glandular, multicellular trichomes [6], especially in desert plants. Therefore, a systematic understanding of the development and function of trichomes in desert plants is worthy of further research. In the present study, we investigated the morphogenesis, cell wall composition, and the preliminary function of the trichomes in *S. ferganica.*

### Non-glandular trichomes of *S. ferganica* are multicellular and uniseriate with 1–2 enlarged basal cells connected with the epidermis

Trichomes represent an excellent model for studying the mechanisms of cell wall biogenesis and development [37]. The morphogenesis of the trichomes in *S. ferganica* began from the epidermal cell, which underwent a longitudinal extension followed by 2–3 rounds of cell division and a final elongation of the top cell, resulting in the formation of a multicellular, unbranched, long trichome. The regulation of cell fate requires a balance between cell proliferation, differentiation, intercellular communication, and morphogenesis control, which are involved in the formation of trichomes [9]. This developmental pattern is different from that in *A. thaliana* and *G. hirsutum*. Cotton fibres are linear extensions that do not involve complex cell division [10]. *Arabidopsis* trichomes produce branches and elongated stalks [38], and each trichome undergoes cell morphogenesis, resulting in precise architecture [6, 11]. In our study, the multicellular trichomes of *S. ferganica* were found to have enlarged and arch-shaped bases, long straight stalks with internodes, and coiled tips, which may be significant for water absorption and retention (unpublished data). Environmental factors play an important role in the regulation of trichome initiation, growth, and development [20, 39]. Among the three plants studied, the similarity was that the trichomes were initiated from the epidermal cells of the leaves or ovules, and the difference was that the trichomes of *S. ferganica* were multicellular and unbranched, compared with the single-cell, branched trichomes observed in *Arabidopsis* or the single-cell, unbranched, and elongated trichomes in cotton. These data suggest that trichomes of different species possess diverse morphology and structures which may confer different functions.

### The cell wall composition of *S. ferganica* trichomes varies dynamically in the development and differs from *Arabidopsis* and cotton

**Fig. 9:**
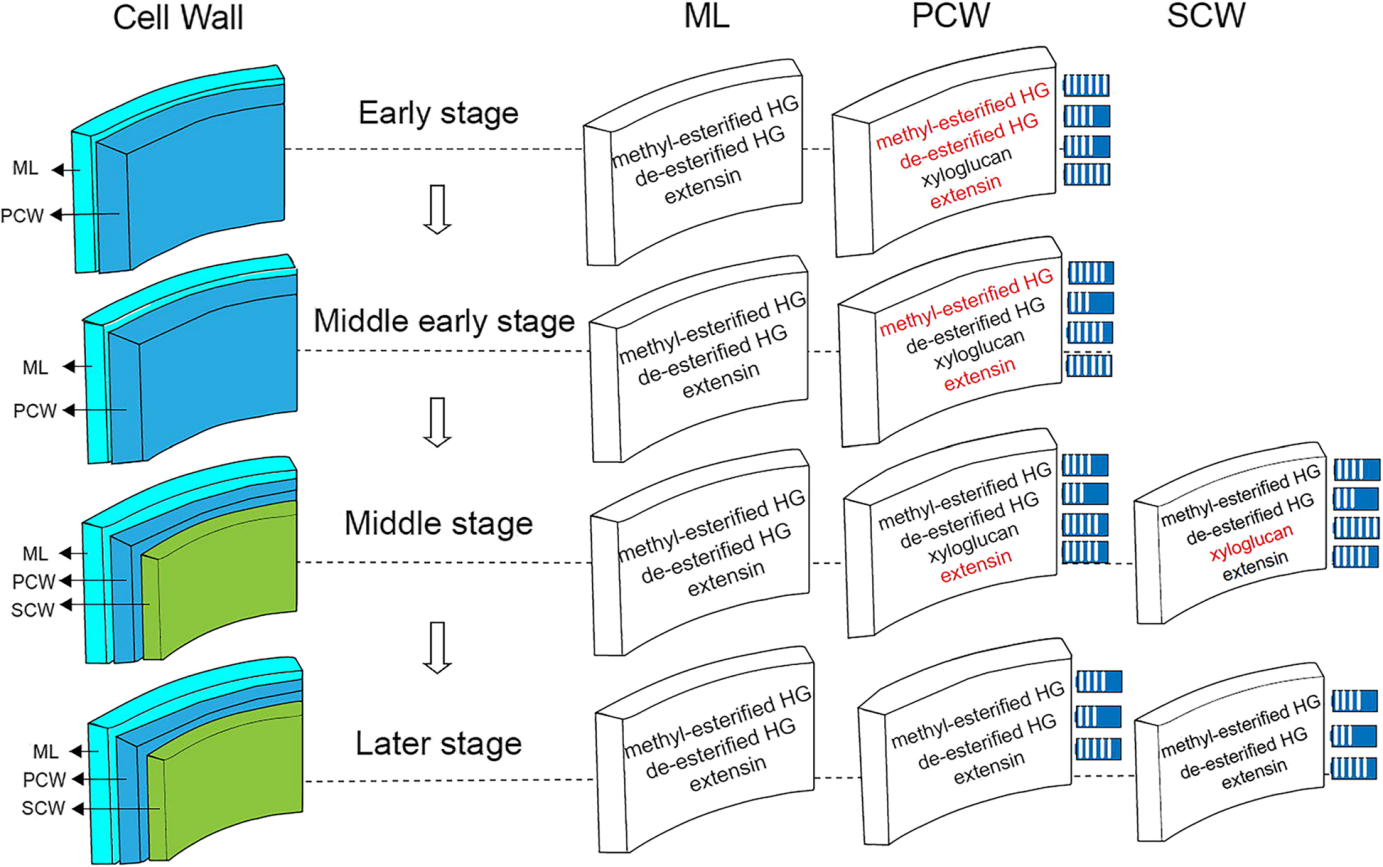
A proposed model for the dynamic changes in the cell wall composition during the trichome development in *S. ferganica*. Polysaccharide components are marked on the separate layers of the cell wall; the black font indicates moderate content of the component; the red font indicates higher content of the component. The blue box with white vertical lines on the right of the primary cell wall (PCW) or secondary cell wall (SCW) layer indicates the intensity of antibody labelling. ML: middle layer

In our study, the cell wall composition of the trichomes of *S. ferganica* was determined, which indicates a special localisation and accumulation of pectins, XGs, and extensin. Generally, the primary cell wall (PCW) contains various polysaccharides, including homogalacturonan (HG), rhamnogalacturonan I (RGΙ), XG, and glycoproteins [40], and the biosynthesis and differentiation of cell walls in plants are controlled by a precise spatial, temporal, and developmental process [19]. Trichome cell walls usually consist of various polysaccharides, which act synergistically to form unique structures [2]. In catchweed bedstraw (*Galium aparine* L.), trichomes are enriched with HGs and RGIs but not XG [13]. In the present study, the immunohistochemical results revealed that highly esterified HGs and de-esterified HGs were abundant in trichomes at the early stage and reduced at the later stage in *S. ferganica*, accompanied by the high accumulation of extensin. XG was significantly enhanced at the mid-early stage and was concentrated in the basal cells of the trichomes at the later stage, whereas the amount of partially methyl-esterified HGs and RGΙs was much lower. The results of this study suggest significant differences in the cell wall components of trichomes among *S. ferganica*, *Arabidopsis*, and cotton (Table 1). Based on the results of the present study and previous reports, we proposed a model for the dynamic change in the cell wall composition of the trichomes from initiation to maturation in *S. ferganica* (Fig. 9). In the early and middle developmental stages, the PCW of trichomes was synthesised and deposited. Polysaccharide components gradually increased, including abundant methyl-esterified pectin, moderately de-esterified pectin, and significantly higher extensin. From mid-early to later stages, components of the SCW were synthesised and deposited on the inner surface of the PCW, the XG was significantly accumulated at this stage. Meanwhile, pectin and extensin were gradually decreased in the PCW.

Plant cell wall contributes to structural integrity, cell adhesion, and defence response mediation during plant growth and development [12]. For example, XGs provide mechanical strength and the dynamics of elastoplasticity to the cell wall [17, 41, 42]; extensin is known to be the key component responsible for rigidification, cross-linking, and stabilisation of cell walls [43]. In the present study, abundant pectin was detected in trichomes of *S. ferganica* before maturation, and XG was significantly increased from the mid-early to middle stages. The accumulated pectin and XG may enhance the PCW and SCW, respectively of the cell wall of trichomes in *S. ferganica*. Previous studies have reported that the hooked trichome of catchweed bedstraw contains a low amount of XG, enabling the formation of a hook-like structure [13]. Additionally, cell wall composition includes extensin that is considered to act as a scaffold for proper wall assembly under biotic or abiotic stress [18, 44]. In the present study, the trichomes of *S. ferganica* distinctly accumulated extensin during development until maturation, especially at the onset of trichome morphogenesis, even though the RGI, AGP, and LMHG were absent from these trichomes. These results suggest that the cell wall of the trichomes of *S. ferganica* is constructed by a specific combination of various polysaccharides, which are different from those of *Arabidopsis* and cotton. 

### Trichome morphogenesis of *S. ferganica* is regulated by trichome-related genes

The expression patterns of some trichome-related genes were analysed to confirm the biogenesis of trichomes [45]. Compared to unicellular trichomes, studies on multicellular trichomes in plants are still limited. In *A. thaliana*, *GL2*, *TTG1*, and *GIS* genes are required for trichome morphogenesis, including cell expansion, branching, and maturation [32, 33, 46]. The microtubule-related genes, *TUBULIN* and *KCBP*, mainly label microtubule arrays that control the early development of trichomes [47, 48]. Proper organisation of F-Actin is essential for normal trichome morphogenesis in *Arabidopsis*, possibly to attract microtubules to the tips of growing branches of the trichomes [46, 49]. ADF proteins are considered to be key modulators of the dynamic organisation of the actin cytoskeleton [50], and FIMBRIN is a cytoskeletal protein associated with microfilaments [51]. Based on information from previous studies, we explored the gene expression patterns in trichomes in *S. ferganica* and found that *α-TUBULIN* and *KCBP* were upregulated in the early stages. In particular, the expression level of *GL2* was the highest in the early stage of the trichome development, whereas it was very low in the cotyledon; hence, it may be critically involved in the initial development of trichomes. Meanwhile, the expression of *F-ACTIN*, *ADF*, and *TTG1* was high in the mature leaves and low in the cotyledons and during senescence. The data suggest that *GL2*, *α-TUBULIN*, *FIMBRIN*, and *KCBP* may be mainly involved in early trichome initiation, whereas *TTG1, GIS*, *F-ACTIN*, and *ADF* may be involved in the elongation of *S. ferganica* trichomes in later stages. Furthermore, *KIC* negatively regulates trichome development in *A. thaliana* [30]. In *S. ferganica*, the expression of *KIC* was low in the early stage and was mainly expressed in the middle and lower parts of mature and senescent leaves. These results suggest that *KIC* may not participate in the early development of trichomes. In addition, the vesicle transport activity was detected in any tissue; *GNL1* is required for vesicle trafficking, showing that its cellular roles are correlated with cell growth [29]. The results of this study showed that *GNL1* was actively expressed in the early and late stages and in the cotyledons of *S. ferganica*. The *WOX3* gene controls trichome development in rice [34]. In *S. ferganica*, *WOX3* expression level increased in the early and late stages, similar to that in cotyledons, therefore, its role in trichome development of *S. ferganica* needs more experiments to clarify. The documentation on trichome morphogenesis and related gene expression should contribute to the further analysis of functions of the trichomes [21, 52]. 

### Trichome length of *S. ferganica* in early morphogenesis can be altered by salt or drought stress

The trichomes are involved in salt stress response and play a critical role in the adaptability and successful survival of plants under harsh environments [53]. *Ocimum basilicum* responds to saline environments by increasing the density of non-glandular trichomes [22]. In the present study, we found that the trichome length of *S. ferganica* was altered under salt and drought stress at the early stage of morphogenesis, i.e., lower concentrations of NaCl promoted growth, higher concentrations inhibited growth; however, lower concentrations of PEG significantly decreased the length of trichomes. The length of the root hairs of *Brassica napus* increases in response to salinity stress [23]. Our findings suggest that trichomes of *S. ferganica* can respond to stimuli and adjust their developmental mode to adapt to environmental changes.

### Trichomes of *S. ferganica* may accumulate and secrete salt to gain tolerance

Halophytes also have specialised trichomes that function in salt accumulation and secretion to gain tolerance, which allows them to grow in highly saline environments [24, 54]. In the present study, crystals or a crust layer on the trichomes of *S. ferganica* were observed under NaCl stress, and the contents of Na^+^ and Cl^−^ were significantly higher in trichomes than those in mesophyll cells. Previous studies have shown that the cubic crystal deposits on leaf surfaces may indicate salt excretion from specific salt secretory structures [55], which plays a role in the maintenance of cellular ion balance (sodium and/or potassium) [56]. The halophyte *Oryza coarctata* (*Porteresia coarctata*) secretes salt through trichomes [24]. Our results suggest that trichomes in *S. ferganica* may accumulate salt from mesophyll cells and secrete it to the external environment. Jahromi et al. (2019) described a subepidermal salt-storage layer (SSL) in *Salsola crassa*, and *S. ferganica* appears to have similar layers (Fig. 3I); the palisade chlorenchyma has evolved into an SSL beneath the leaf epidermal layer [57]. This also indicates that *Salsola* species may have redundant pathways and mechanisms to perform the same function [54]. Generally, ion homeostasis can be re-established under salt stress that helps the plant cells to adapt to adverse conditions [58]. *Aeluropus lagopoides* has an adaptive character that allows it to thrive in harsh desert conditions by excretion of excessive toxic ions and retention of K^+^ in the trichomes [53]. In our study, the K^+^ levels in trichomes of *S. ferganica* were similar to that in the mesophyll cells under higher salt concentrations, suggesting that the trichomes of *S. ferganica* may selectively accumulate ions. All these changes are possibly evolutionary strategies developed by the *S. ferganica* plants that allow them to grow well in saline soils.

## Conclusions

Plants have evolved special accessory structures to survive in harsh environmental conditions and combat stress. Trichomes in plants are specialised structures that play a major role in interaction with environments. Thus, evaluating the trichome biogenesis and functions can help us understand the stress tolerance mechanism in desert plants. The results indicate that *S. ferganica* has multicellular, non-branched trichomes that undergo two to three rounds of cell division and are affected by abiotic stress. They have a unique cell wall composition that is different from that of the trichomes of *A. thaliana* and cotton. Furthermore, several genes are positively or negatively involved in the regulation of the trichome development of *S. ferganica*. Additionally, we preliminarily examined the function of salt accumulation in the trichomes of *S. ferganica*. These results suggest that the desert plant *S. ferganica* has unique trichome morphology, structures and functions, which are closely related to their survival environment. 

## Materials and methods

### Plant cultivation and treatments

Seeds of *S. ferganica* were collected from mature plants in their natural habitat in 2020 at the Wujiaqu 103 regiment (44°37′N, 87°26′E; 423 mH), Xinjiang, China. The *S. ferganica* specimens were identified by Yongman Lu, who is a plant taxonomist in College of Life Science and Technology, University of Xinjiang. The voucher specimen was deposited in the herbarium (College of Life Science and Technology, Xinjiang University) with voucher No: SF200930. After air-drying indoors and cleaning, the seeds were stored in brown paper bags at 4 °C for future uses.

To observe the trichome developmental process, determine the polysaccharides in trichome cell wall, and perform quantitative real-time PCR (qRT-PCR) in the early stage, seeds were soaked for 5 min in the corresponding solutions (100, 300 mmol·L^− 1^ NaCl; 10% polyethene glycol (PEG) 6000), and then incubated on two layers of filter paper saturated with the above solutions in Petri dishes under a 16 h light (25 °C)/ 8 h dark (22 °C) regime in a plant cultivation incubator for 2 weeks. Samples were collected for qRT-PCR from 24 h to 3 d at intervals of 4–8 h (until trichomes could be seen with the naked eye) after being sown, or when young leaves emerged at 24 h, 36 h, 60 h, 72 h, 96 h, and 144 h. Further, cotyledons were sampled at 12 h and 72 h after being sown for qRT-PCR analysis.

To perform qRT-PCR in the later stage, seeds were pre-treated with H_2_O at 37 °C for 12 h, sown in pots containing soil matrix (perlite: vermiculite = 1:3, v/v), and cultivated for approximately 2 months under the following conditions: 20–30 °C, 14–16 h light/8–10 h dark, 10–30% relative humidity (RH). After the first 2 weeks, half-strength Hoagland solution [59] was applied at intervals of 2 weeks. Leaves were sampled from the four-leaf and eight-leaf seedlings; from the top, upper, middle, and lower parts; and the whole adult plant. Trichomes were sampled only from naturally senescent leaves gathered from approximately 3-month-old plants. For the element distribution assay of trichomes, 4–6-week-old seedlings from the pot cultivation were treated with different concentrations of salt solution (0, 100, 300, 500 mmol·L^− 1^ NaCl, prepared in 1/2 Hoagland solution) for 2 weeks and then sampled.

To observe trichome development and determine trichome cell wall polysaccharides in *A. thaliana* and *G. hirsutum* (as controls), we sowed seeds of *A. thaliana* (Columbia-0) on 1/2 MS medium plates and vernalised for 48 h at 4 °C in the dark. The seed plates were maintained under the following conditions: 16 h light (25 °C)/8 h dark (22 °C), 60–80% RH, 120–150 μmol∙m^− 2^∙s^− 1^ of light intensity. True leaves were collected daily from the first day of germination to the 15th day. On the 30th day, *A. thaliana* seedlings selected from the above seed plates were transferred into pots containing a mixture of nutrient soil:vermiculite = 3:1 (v/v) and grown under similar conditions (except for 30% RH) until bolting. *G. hirsutum* (‘Tezaonan 2’) was grown in the trial plots outdoors with regular water and fertiliser supply until blooming. The cotton ball was sampled every day from the 3 DBA to the 20 DPA of blooming.

### Observation of the morphology and microstructure of *S. ferganica* trichomes

Trichome biogenesis and development were visualised under a stereomicroscope (SMZ25; Nikon, Japan). The morphology and microstructure of the trichomes were inspected using a LEO-1430VP scanning electron microscope (SEM; Carl Zeiss, Germany) and a Quanta 200 environmental scanning electron microscope (ESEM; Eindhoven, Netherlands). The length and density of the trichomes were measured using ImageJ software (NIH ImageJ system, Bethesda, MD, USA). Three biological replicates of each treatment and three to five observations of each sample were applied.

### Observation of the cytological process of trichome development and the localisation of cell wall polysaccharides

The true leaves of *S. ferganica*, *A. thaliana*, and *G. hirsutum* were collected during different developmental periods. Plant tissues were fixed in FAA solution (90 mL 50% ethanol, 5 mL formaldehyde, and 5 mL glacial acetic acid) for paraffin sectioning. Subsequently, the samples were quickly and gently vacuumed repeatedly until the tissues dropped to the bottom of the bottle, which were then incubated at 4 °C for 24 h. After fixation, tissues were stained in 1% safranine-O for 8–10 h, and then dehydrated with different concentrations of ethanol and xylene. For solidification and paraffin inclusion, tissues were successively treated with first, second, and third grade paraffin. The melted paraffin-embedded tissues were poured into a paper tank and quickly placed into iced water to solidify for paraffin embedding. Following this, paraffin blocks containing tissues were sliced into 6–12 μm sections using a microtome (Leica RM2126), and sections were expanded and deparaffinised using successive xylene and ethanol treatments. For safranine-fast green counterstaining, tissues on the slide were stained in 1% safranine-O overnight, quickly and gently dipped into 1% fast green for approximately 10 s, and immediately treated with a series of ethanol and xylene solutions.

For immuno-histochemical localisation, the sections prepared using the above method were spread on siliconised slides to prevent them from dropping off. The endogenous peroxidase was inactivated by incubation with 0.3% H_2_O_2_ for 20 min and then the samples were rinsed three times with 0.01 mol·L^− 1^ phosphate-buffered saline (pH 7.2–7.4; PBST; Cat. P1010, Solarbio, China; containing 0.05% Tween-20) for 5 min. A heated citrate buffer was used for antigen retrieval, and the blots were rinsed three times with PBST buffer for 5 min. Following this, slides were blocked with 1% bovine serum albumin (BSA) for 30–60 min and diluted with 1% BSA primary antibodies (1:10) of JIM5, JIM7, CCRC-M1, CCRC-M7, and CCRC-M38 (Carbosource Services, GA, USA), and LM1 (PlantProbes, Leeds, UK) to recognise different cell wall polysaccharides. After incubation at 37 °C for 1 h, the slides were rinsed five times with PBST buffer for 10 min, and then allowed to react with horseradish peroxidase (HRP)-labelled secondary antibody (1:500 diluted with 1% BSA) at 37 °C for 1 h. Sections were incubated with diaminobenzidine (DAB) (Sangon, Shanghai) for 7 min to develop the colour and then dehydrated in absolute ethanol for 5 min (two changes) and xylene for 5 min (two changes). For the controls of the immunohistochemical analysis, primary antibodies were omitted, and the sections were incubated with secondary antibodies only (Fig. S8).

Neutral gum and a cover slip were added to all sections, and they were sealed with nail polish for mounting. Finally, the prepared sections were inspected under a light microscope (Nikon ECLIPSE Ti-E, Japan), and photographs were taken using Nis-Elemens software (Japan).

### Quantitative RT-PCR (qRT-PCR)

Total RNA was extracted from *S. ferganica* seedlings using a Plant RNA Kit (R6827–02, Omega, USA). Reverse transcription was performed using M-MLV (TaKaRa, Dalian, China) according to the manufacturer’s protocol. qRT-PCR was performed in ABI 7500 Real-time PCR system (Applied Biosystem, USA) using PerfectStart Green qPCR SuperMix Kit (Cat. AQ601–02; Transgen, China), and *β-ACTIN* was used as an internal reference. Eleven trichome-related genes based on reported sequences were selected: *α-TUBULIN*, *F-ACTIN*, *FIMBRIN*, *KCBP*, *ADF*, *GNL1*, *KIC*, *GL2*, *GIS*, *TTG1*, and *WOX3*. Coding regions of these genes from other species of Chenopodiaceae were aligned; degenerate primers were designed to obtain short DNA sequences of each gene from *S. ferganica*, and then accurate qRT-PCR primers were designed based on the acquired short sequences of each gene (Table S1). The PCR was performed under the following conditions: 95 °C for 2 min followed by 40 cycles at 95 °C for 5 s and at 60 °C for 30 s. Relative quantification of specific mRNA level was calculated using the cycle threshold (CT) 2^-ΔΔCT^ method (Shi and Chiang, 2005), where ΔΔCT = ΔCT_target sample_ − ΔCT_control sample_, ΔCT_target sample_ = CT_test gene_ − CT_reference gene_. Three biological replicates with three technical replicates each were performed for each treatment. The final results were expressed as the fold change of the target sample compared with the control value at 24 h.

### Analysis of the element distribution in trichomes under NaCl treatment

The elemental distribution in the trichomes and epidermal layer was examined using energy dispersive spectroscopy (EDS, Oxford 7353, Oxford, United Kingdom). Plants treated with different concentrations of NaCl (0, 100, 300, and 500 mmol·L^− 1^) were used. The middle segment of the first fully expanded leaf of each plant was measured, and at least three leaves were sampled for each biological replicate. All samples were collected and measured between 11:00 AM–01:00 PM, inactivated at 105 °C for 30 min, and dried at 85 °C for 12 h. The fleshy leaves were then cut open with a razor blade. Subsequently, the transverse sections of the mesophyll and trichome cells were scanned, and the mass percentages of Na^+^, Cl^−^, Mg^2+^ and K^+^ in trichomes and epidermal cells were determined. Three biological replicates were performed for each treatment.

### Statistical analysis

All data are expressed as mean ± standard deviation (SD). The comparison of unpaired data between two groups was conducted using an unpaired *t*-test. Data among multiple groups were compared using one-way analysis of variance (ANOVA) followed by Tukey’s test using GraphPad Prism 5.0 for Windows (GraphPad Software, San Diego, CA, USA). Significant differences were analysed by multiple comparisons at *P* values of 0.05, 0.01, or 0.001 levels.

## Supplementary Information

**Additional file 1: Table S1.** Primer sequences used in the present study. **Table S2.** A detail description of the polysaccharide recognition epitopes in *S. ferganica*. **Fig. S1.** Morphology of cotyledons at different stages in *S. ferganica*. **Fig. S2.** Morphology and cytological structures of trichomes during the seed or leaf development in *G. hirsutum* or *A. thaliana*, respectively. **Fig. S3.** Cellular localisation of low methyl-esterified (0–30%) homogalacturonan (LMHG) (recognised by JIM5) in the trichomes of *S. ferganica*, using *Arabidopsis* and cotton as controls (the same below). **Fig. S4.** Localisation of heavily methyl-esterified (50–100%) homogalacturonan (HMHG) (recognised by JIM7) in the trichome of *S. ferganica*. **Fig. S5.** Localisation of fully de-esterified HG (FDEHG) (recognised by CCRC-M38) in the trichome of *S. ferganica*. **Fig. S6.** Localisation of rhamnogalacturonan I (RGI) and arabinogalactan glycoprotein (AGP) (recognised by CCRC-M7) in the trichomes of *S. ferganica*. **Fig. S7.** Localisation of xyloglucan (XG) (recognised by CCRC-M1) in the trichomes of *S. ferganica*. **Fig. S8.** Negative controls of immunohistochemical assay in *S. ferganica*. **Fig. S9.** The morphology of *S. ferganica* under treatment with different concentrations of PEG 6000. **Fig. S10.** Effect of drought stress on trichome development in *S. ferganica*.

## Data Availability

The dataset generated during and/or analysed during the current study is available from the corresponding author upon reasonable request.
