# Supplementary material for: Morphogenesis and cell wall composition of trichomes and their function in response to salt in halophyte Salsola ferganica
Source: BMC Plant Biol. 2022 Nov 30;22:551. doi: 10.1186/s12870-022-03933-x (PMC9710055; doi:10.1186/s12870-022-03933-x)
Supplement: Supplementary file 1 — Additional file 1: Table S1. Primer sequences used in the present study. Table S2. A detail description of the polysaccharide recognition epitopes in S. ferganica. Fig. S1. Morphology of cotyledons at different stages in S. ferganica. Fig. S2. Morphology and cytological structures of trichomes during the seed or leaf development in G. hirsutum or A. thaliana, respectively. Fig. S3. Cellular localisation of low methyl-esterified (0–30%) homogalacturonan (LMHG) (recognised by JIM5) in the trichomes of S. ferganica, using Arabidopsis and cotton as controls (the same below). Fig. S4. Localisation of heavily methyl-esterified (50–100%) homogalacturonan (HMHG) (recognised by JIM7) in the trichome of S. ferganica. Fig. S5. Localisation of fully de-esterified HG (FDEHG) (recognised by CCRC-M38) in the trichome of S. ferganica. Fig. S6. Localisation of rhamnogalacturonan I (RGI) and arabinogalactan glycoprotein (AGP) (recognised by CCRC-M7) in the trichomes of S. ferganica. Fig. S7. Localisation of xyloglucan (XG) (recognised by CCRC-M1) in the trichomes of S. ferganica. Fig. S8. Negative controls of immunohistochemical assay in S. ferganica. Fig. S9. The morphology of S. ferganica under treatment with different concentrations of PEG 6000. Fig. S10. Effect of drought stress on trichome development in S. ferganica. [file 12870_2022_3933_MOESM1_ESM.docx]

**Table S1** Primer sequences used in the present study

| Gene | Primer sequence (5’-3’) | |
| --- | --- | --- |
|  | Forward | Reverse |
| *ADF* | TGACACTGCGAGGGTGAGGAG | TGAAGTTCCACCTGAATGCCATCC |
| *α-TUBULIN* | CCAACTGGTTTCAAGTGTGG | CGATTCTCCCAAATACTTCAGC |
| *F-ACTIN* | GCATCCAGGCTCAGTCCTAA | TGATGTTGGTTATGGCTACAGC |
| *FIM* | TTCAGCATAGGAATGGGCTCT | CTAAGAGTATCCATCCATCTCGGA |
| *GIS* | GGAGATTCGAGTGTCACTATTGTT | CGTTTGTGGGCGTTTTGGTG |
| *GL2* | CAGATGAGAAGCAAAGGCAAC | GGACTTGTTCTCCTCTTTCACCT |
| *GNL1* | AATGGTGCTGGTGCTCACACTAAC | GCTGATGTCGGCTGTCTCATGTC |
| *KCBP* | GTGATGCTGGAACTGAGGAGTA | AAACATTGGGTCAGTCACAGC |
| *KIC* | ACGTCCGATAGCCTGAGGAAGAAC | GGCTAAGCCTCACCATCAGAATGC |
| *TTG1* | GGTTTACGACATTGCTTGGG | ACCTCACATCTTGCTTGTTCC |
| *WOX3* | GGGGAGGGAAGGAAGAAGAAAAGG | CCACATACCACCACCACCACTTC |
| *β-ACTIN* | TCTACAATGAGCTTCGTGTGGC | CACCATCACCAGAATCCAGCAC |

**Table S2** A detail description of the polysaccharide recognition epitopes in *S. ferganica*

| Antibody | Recognition | *Salsola ferganica* | *Gossypium hirsutum* | *Arabidopsis thaliana* |
| --- | --- | --- | --- | --- |
| Pectins | | | | |
| JIM5 | Highly de-esterified homogalacturonans | No or very weak reaction | All cells but epidermis 1 DBA, pectin sheath at 0-2 DPA | The main labeling was in mesophyll cells and trichomes cells, and there was almost no labeling in epidermal cells |
| JIM7 | Highly esterified homogalacturonans | Strong reaction in epidermis throughout stages, low levels in the early stages | Strong reaction in epidermis throughout stages | Weak labeling in mesophyll cells, more intense labeling in trichomes cells and epidermal cells |
| CCRC-M38 | Pectic mucilage, de-branched RGI | Less trichomes cells than other tissue cells | Strong reaction except in fiber cell cross-walls, pectin sheath | It is evenly labeled in the mesophyll cell wall and trichomes |
| AGPs | | | | |
| CCRC-M7 | AGP/RGI epitope | No or very weak reaction, Mature true leaf tissue (except trichomes) cells are responsive | Strong on epidermis, lost from fiber cells at 1-2 DPA | The labeling was very obvious in trichomes cells and mesophyll cells |
| Xyloglucans | | | | |
| CCRC-M1 | Fucosylated xyloglucans | Trichomes tissue is higher than other tissues, with a gradual decrease in response in the later stages | Uniform reaction throughout tissue | There was almost no labeling in mesophyll cells, and it was weakly and evenly distributed in trichomes cells and epidermal cells |
| Extensin | | | | |
| LM1 | Extensin | Trichomes cells are significantly more abundant than other plant tissues, but the content decreases with time | No or very weak reaction  Trichomes cells are slightly more abundant than other tissues | Trichomes cells are weakly distributed relative to mesophyll cells |


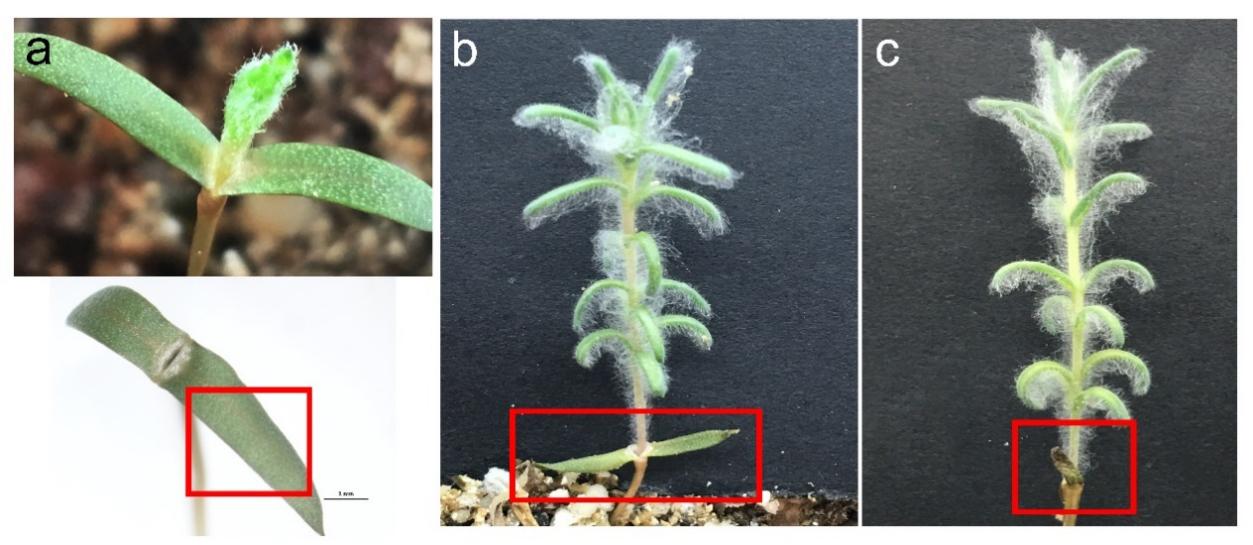


**Fig. S1** Morphology of cotyledons at different stages in *S. ferganica*. Cotyledons (marked in red boxes). **a** 20 d. **b** 45 d. **c** 60 d


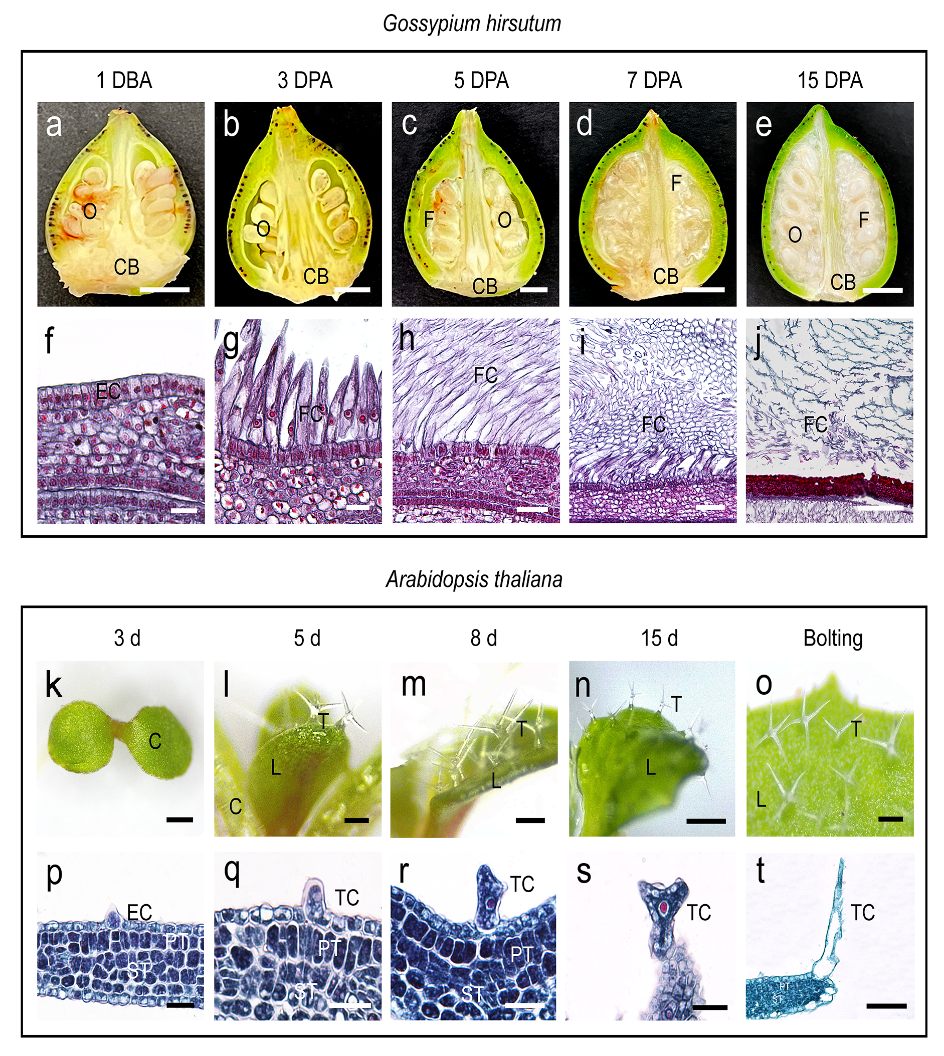


**Fig. S2** Morphology and cytological structures of trichomes during the seed or leaf developments in *G. hirsutum* or *A. thaliana*, respectively. **a–j** *G. hirsutum*. **f**–**j** Cytological structure of ovule fibres corresponding to a–e. **a**, **f** 1 DBA; **b**, **g** 3 DPA; **c**, **h** 5 DPA; **d**, **i** 7 DPA; **e**, **j** 15 DPA. **k**–**t** *A. thaliana*. **p**–**t** Anatomic structures of leaf trichomes corresponding to **k**–**o**. **k**, **p** Germination for 3 d; **l**, **q** 5 d; **m**, **r** 8 d; **n**, **s** 15 d; **o**, **t** Bolting stage. Scale bar: **a**, **b**, **c**, 250 μm; **d**, 500 μm; **e**, 1 cm; **f**, **g**, **p**-**r**, **s**, 25 μm; **h**, **t**, 50 μm. **i**, **l**, **m**, 100 μm. **j**, **k**, **n**, **o**, 200 μm. O, ovule; CB, cotton boll; F, fiber; FC: fibre cell; L: leaf; T: mature trichomes; C: cotyledon; EC: epidermal cell; TC: trichome cell; PT: palisade tissue; ST: spongy tissue; DBA: days before anthesis; DPA: days post anthesis


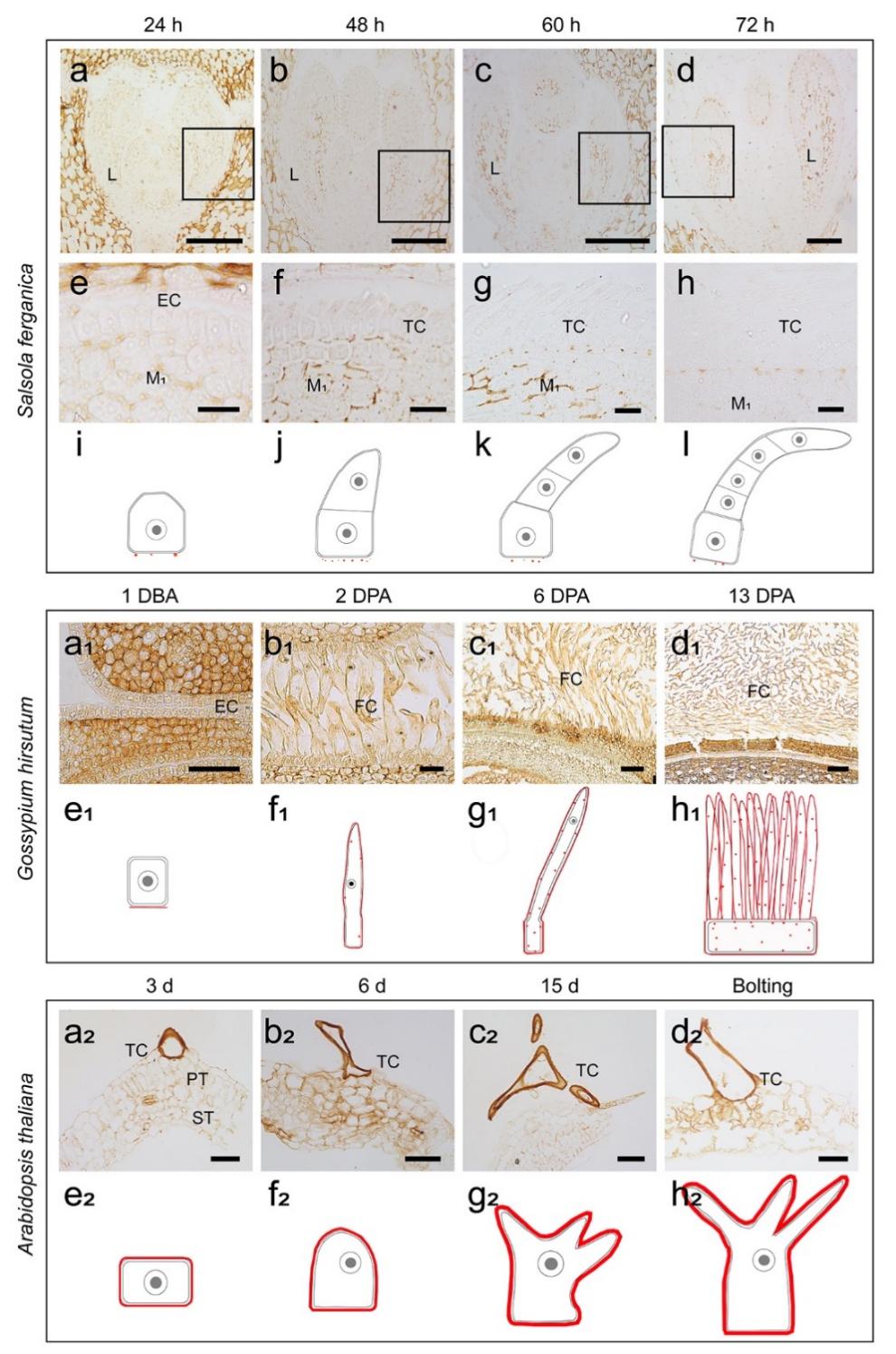


**Fig. S3** Cellular localisation of low methyl-esterified (0–30%) homogalacturonan (LMHG) (recognised by JIM5) in the trichomes of *S. ferganica*, using *Arabidopsis* and cotton as controls (the same below). **a**–**d** LMHG localisation in trichomes at different developmental stages in *S. ferganica*. **e**–**h** Local enlarged views corresponding to a–d. **i**–**l** Hand-drawn diagram of trichomes in the developmental process showing LMHG localisation of *S. ferganica*. **a**, **e**, **i** Germination for 24 ± 12 h; **b**, **f**, **j** 48 ± 12 h; **c**, **g**, **k** 60 ± 12 h; **d**, **h**, **l** 72 ± 12 h. **a_1_**–**h_1_** LMHG localisation in fibres of *G. hirsutum*. **e_1_**–**h_1_** Hand-drawn diagram of fibres in the developmental process showing LMHG localisation. **a_1_**, **e_1_** 1 DBA; **b_1_**, **f_1_** 2 DPA; **c_1_**, **g_1_** 6 DPA; **d_1_**, **h_1_** 13 DPA. **a_2_**–**h_2_** LMHG localisation of trichomes in *A. thaliana*. **e_2_**–**h_2_** Hand-drawn diagram of trichomes in the developmental process showing LMHG localisation. **a_2_**, **e_2_** Germination for 3 d; **b_2_**, **f_2_** 6 d; **c_2_**, **g_2_** 15 d; **d_2_**, **h_2_** bolting. Scale bar: **a**–**d**, 200 μm; **e**–**h,** 25 μm; **a_1_**, **b_1_**, 50 μm; **c_1_**, **d_1_**, 100 μm; **a_2_**–**d_2_**, 40 μm


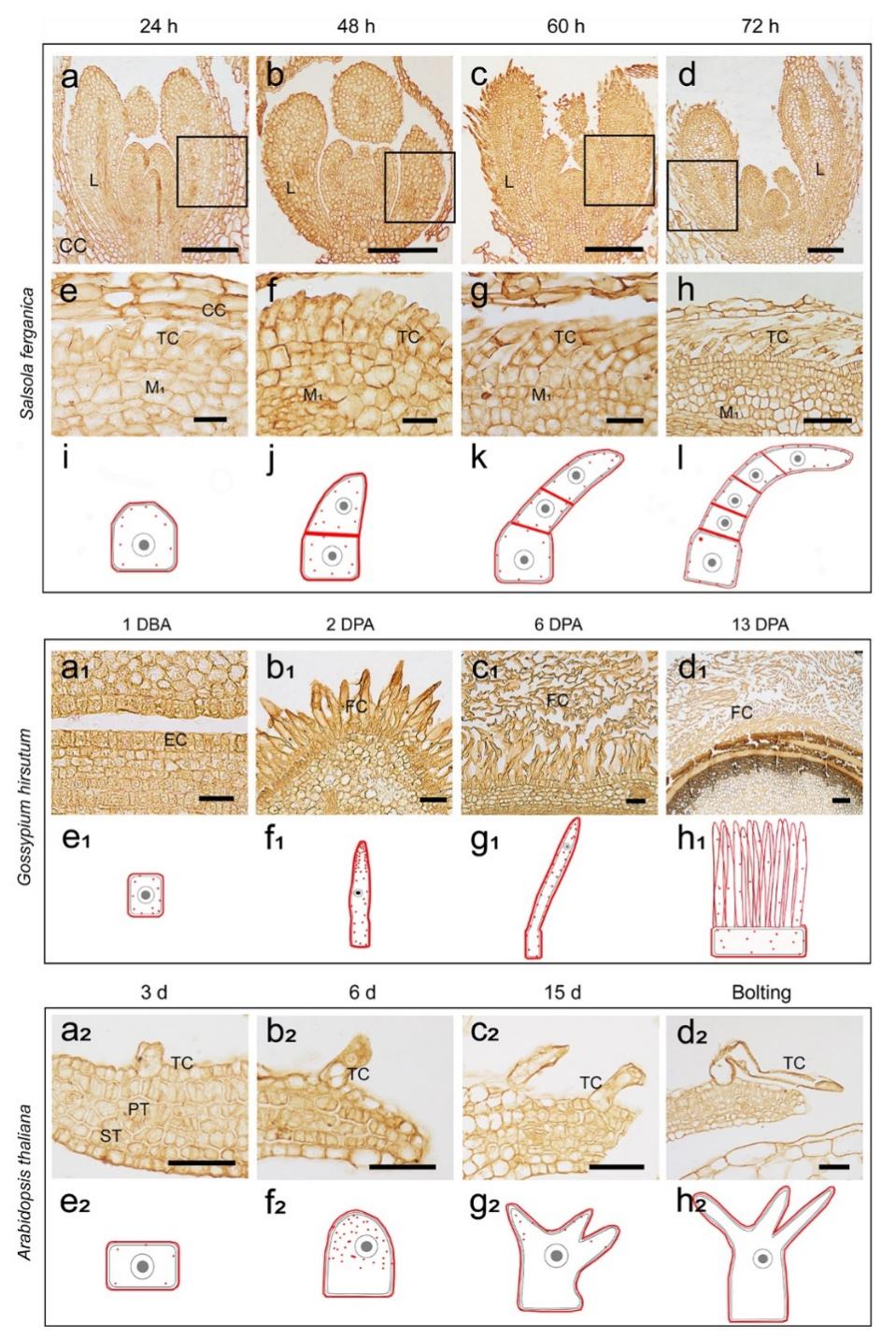


**Fig. S4** Localisation of heavily methyl-esterified (50–100%) homogalacturonan (HMHG) (recognised by JIM7) in the trichome of *S. ferganica*. **a**–**l** HMHG localisation in trichomes at different developmental stages in *S. ferganica*. **e**–**h** Local enlarged views corresponding to a–d. **i**–**l** Hand-drawn diagram of trichomes in the developmental process showing HMHG localisation. **a**, **e**, **i** Germination for 24 ± 12 h; **b**, **f**, **j** 48 ± 12 h; **c**, **g**, **k** 60 ± 12 h; **d**, **h**, **l** 72 ± 12 h. **a_1_**–**h_1_** HMHG localisation in fibres in *G. hirsutum*. **e_1_**–**h_1_** Hand-drawn diagram of fibres in the developmental process showing LMHG localisation. **a_1_**, **e_1_** 1 DBA; **b_1_**, **f_1_** 2 DPA; **c_1_**, **g_1_** 6 DPA; **d_1_**, **h_1_** 13 DPA. **a_2_**–**h_2_** LMHG localisation of trichomes in *A. thaliana*. **e_2_**–**h_2_** Hand-drawn diagram of trichomes in the developmental process showing HMHG localisation. **a_2_**, **e_2_** Germination for 3 d; **b_2_**, **f_2_** 6 d; **c_2_**, **g_2_** 15 d; **d_2_**, **h_2_** Bolting. Scale bar, **a**–**d**, 200 μm; **e**–**g**, 25 μm; **h**, **c_1_**, **d_1_**, 100 μm; **a_1_**, **b_1_**, 50 μm; **a_2_**, **b_2_**, **c_2_**, **d_2_**, 40 μm


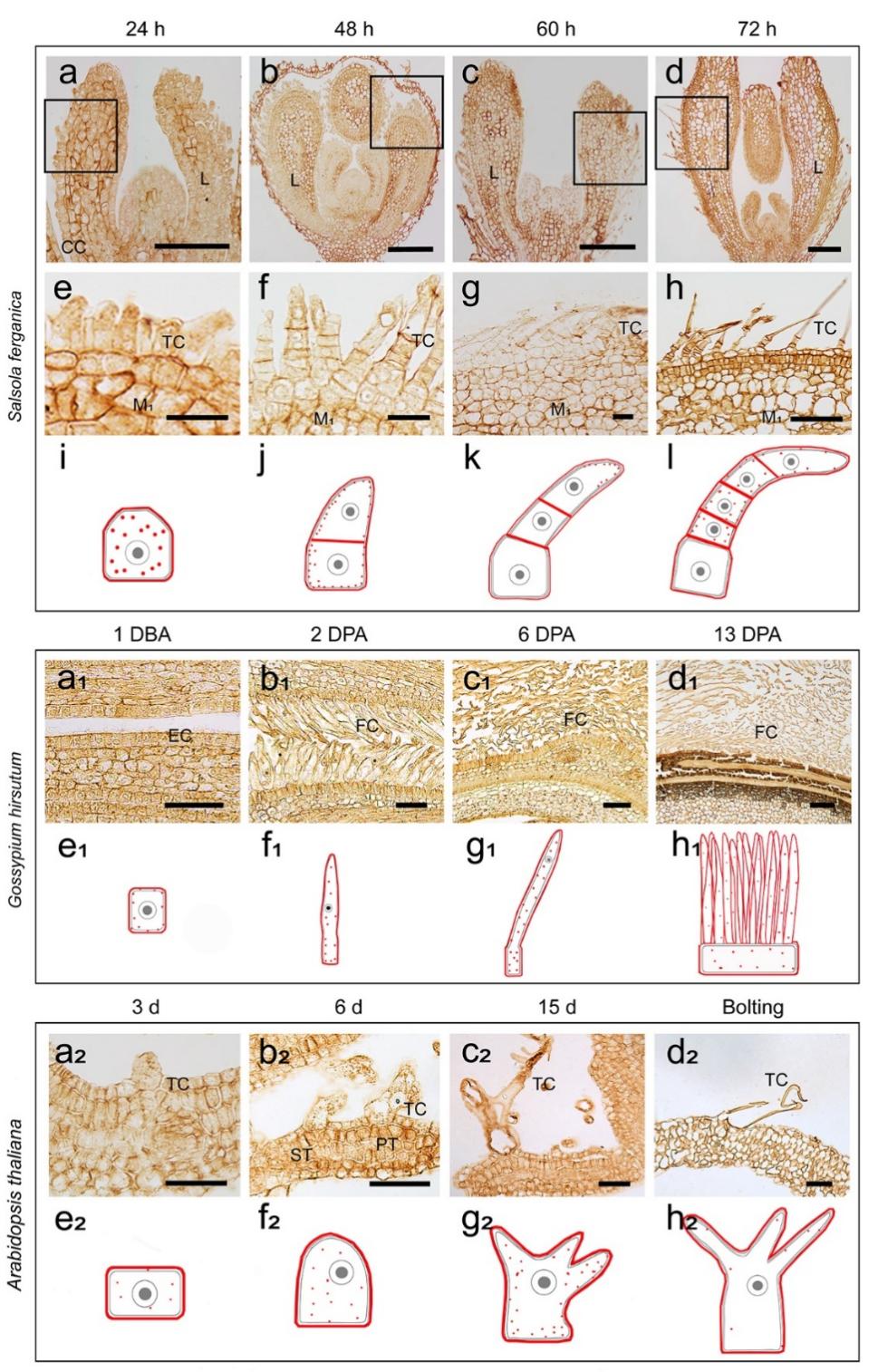


**Fig. S5** Localisation of fully de-esterified HG (FDEHG) (recognised by CCRC-M38) in the trichome of *S. ferganica*. **a**–**l** FDEHG localisation in trichomes of *S. ferganica* at different developmental stages. **e**–**h** Local enlarged views corresponding to **a**–**d**. **i**–**l** Hand-drawn diagram of trichomes in the developmental process showing FDEHG localisation. **a**, **e**, **i** Germination for 24 ± 12 h; **b**, **f**, **j** 48 ± 12 h; **c**, **g**, **k** 60 ± 12 h; **d**, **h**, **l** 72 ± 12 h. **a_1_**–**h_1_** FDEHG localisation in fibres of *G. hirsutum*. **e_1_**–**h_1_** Hand-drawn diagram of fibres in the developmental process showing FDEHG localisation. **a_1_, e_1_** 1 DBA; **b_1_**, **f_1_** 2 DPA; **c_1_**, **g_1_** 6 DPA; **d_1_**, **h_1_** 13 DPA. **a_2_**–**h_2_** FDEHG localisation of trichomes of *A. thaliana*. **e_2_**–**h_2_** Hand-drawn diagram of trichomes in developmental process showing FDEHG localisation. **a_2_**, **e_2_** Germination for 3 d; **b_2_**, **f_2_** 6 d; **c_2_**, **g_2_** 15 d; **d_2_**, **h_2_** Bolting. Scale bar: **a**, **h**, 100 μm; **b**–**d**, **d_1_**, 200 μm; **e**–**g**, 25 μm; **a_1_**–**c_1_**, 50 μm; **a_2_**-**d_2_**, 40 μm


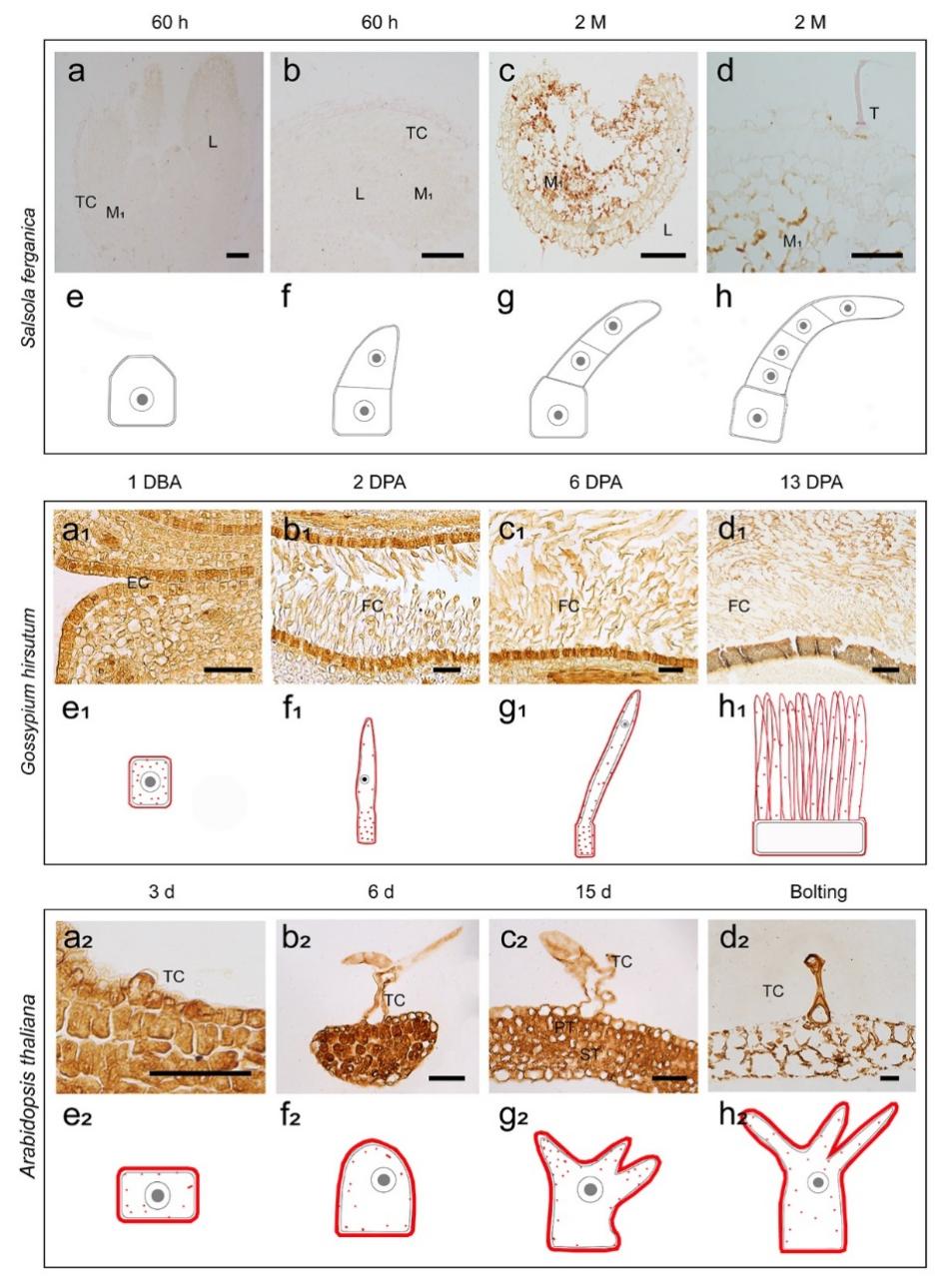


**Fig. S6** Localisation of rhamnogalacturonan I (RGI) and arabinogalactan glycoprotein (AGP) (recognised by CCRC-M7) in the trichomes of *S. ferganica*. **a**–**h** RGI and AGP localisation of trichomes of *S. ferganica* at different developmental stages. **e**–**h** Hand-drawn diagram of trichomes in the developmental process showing RGI and AGP localisation in *S. ferganica*. **a**, **b** Germination for 60 ± 12 h; **c**, **d** 2 M. **a_1_**–**h_1_** RGI and AGP localisation in fibres of *G. hirsutum*. **e_1_**–**h_1_** Hand-drawn diagram of fibres in the developmental process showing RGI and AGP localisation. **a_1_**, **e_1_** 1 DBA; **b_1_**, **f_1_** 2 DPA; **c_1_**, **g_1_** 6 DPA; **d_1_**, **h_1_** 13 DPA. **a_2_**–**h_2_** RGI and AGP localisation of trichomes of *A. thaliana*. **e_2_**–**h_2_** Hand-drawn diagram of trichomes in the developmental process showing RGI and AGP localisation. **a_2_**, **e_2_** Germination for 3 d; **b_2_**, **f_2_** 6 d; **c_2_**, **g_2_** 15 d; **d_2_**, **h_2_** Bolting. Scale bar: **a**, 100 μm; **b**–**d**, **d_1_**, 200 μm; **a_1_**–**c_1_**, 50 μm; **a_2_**–**d_2_**, 40 μm


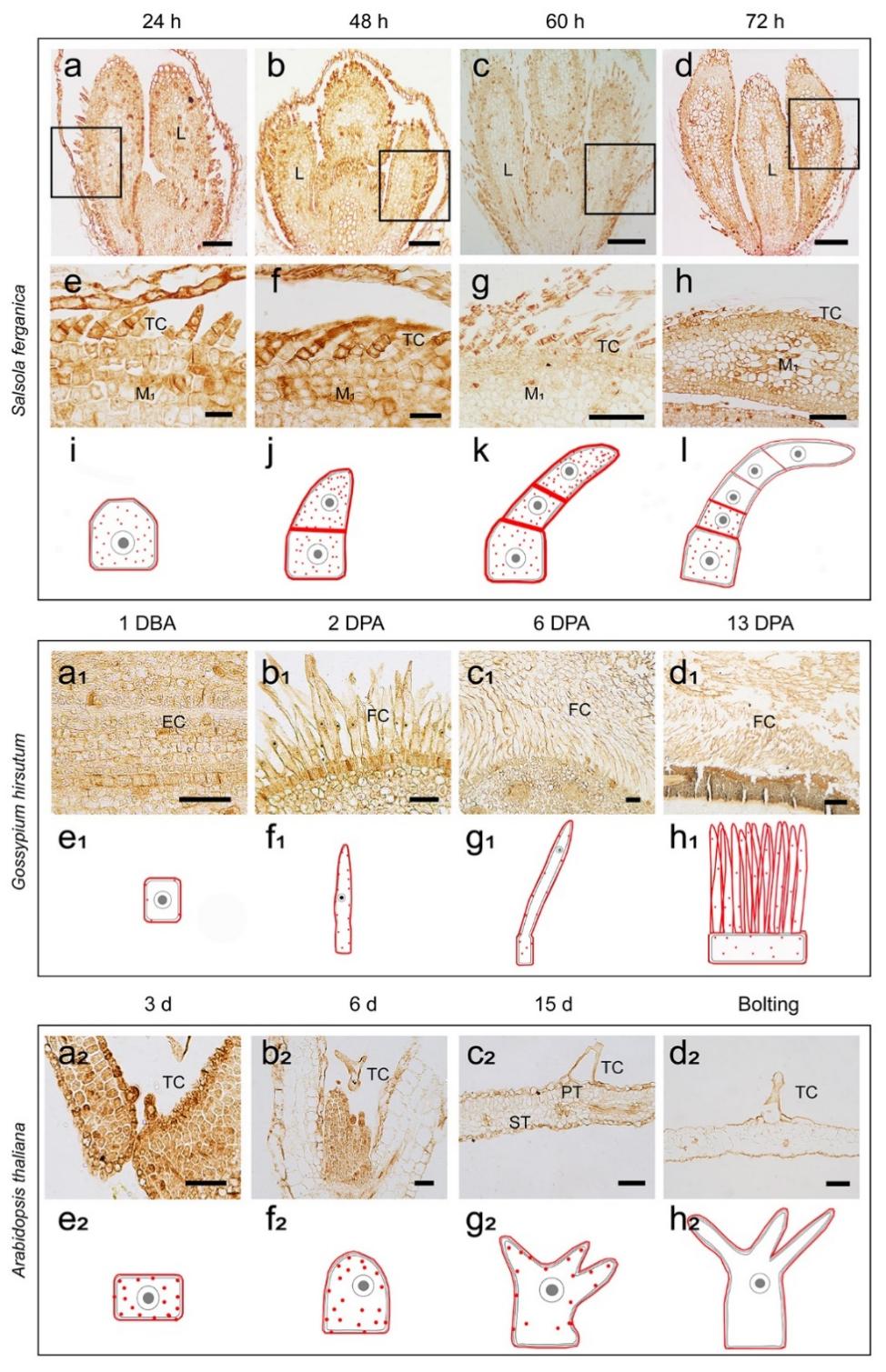


**Fig. S7** Localisation of xyloglucan (XG) (recognised by CCRC-M1) in the trichomes of *S. ferganica*. **a**–**l** XG localisation in trichomes of *S. ferganica* at different developmental stages. **e**–**h** Local enlarged views corresponding to **a–d**. **i**–**l** Hand-drawn diagram of trichomes in the developmental process showing XG localisation. **a**, **e**, **i** Germination for 24 ± 12 h; **b**, **f, j** 48 ± 12 h; **c**, **g**, **k** 60 ± 12 h; **d**, **h**, **l** 72 ± 12 h. **a_1_**–**h_1_** XG localisation in fibres of *G. hirsutum*. **e_1_**–**h_1_** Hand-drawn diagram of fibres in the developmental process showing XG localisation. **a_1_**, **e_1_** 1 DBA; **b_1_**, **f_1_** 2 DPA; **c_1_**, **g_1_** 6 DPA; **d_1_**, **h_1_** 13 DPA. **a_2_**–**h_2_** XG localisation in trichomes of *A. thaliana*. **e_2_**–**h_2_** Hand-drawn diagram of trichomes in the developmental process showing XG localisation. **a_2_**, **e_2_** Germination for 3 d; **b_2_**, **f_2_** 6 d; **c_2_**, **g_2_** 15 d; **d_2_**, **h_2_** Bolting. Scale bar: **a**, **b**, **g**, **h, d_2_**, 100 μm; **c**, **d**, **d_1_**, 200 μm; **e**, **f**, 25 μm; **a_1_**–**c_1_**, 50 μm; **a_2_**–**c_2_**, 40 μm


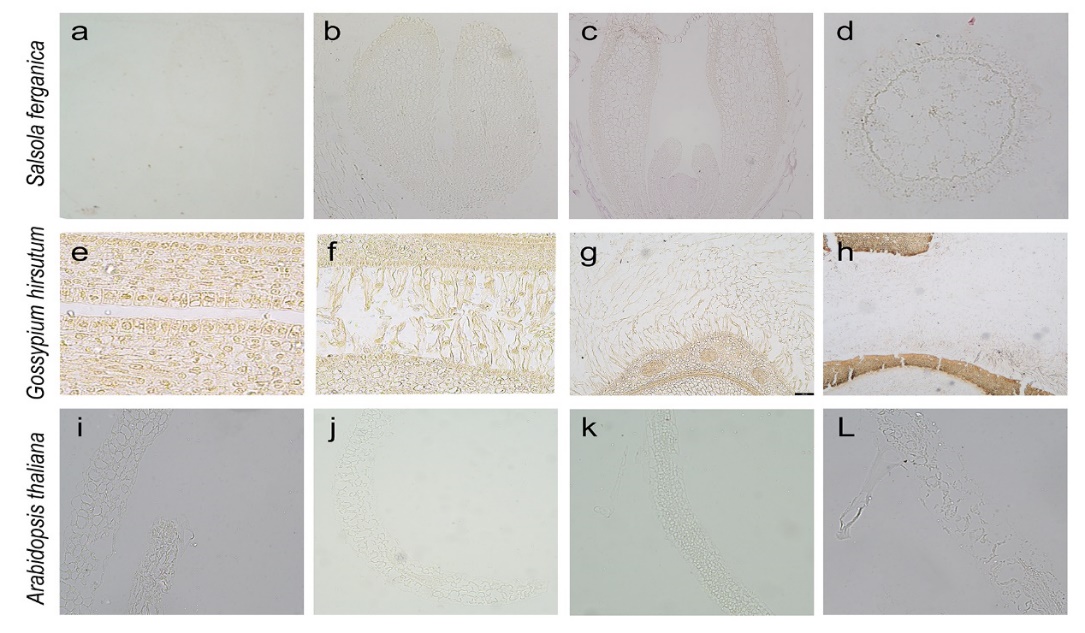


**Fig. S8** Negative controls of immunohistochemical assay in *S. ferganica*. **a–d** *S. ferganica*; **e–h** *G. hirsutum* (1 DBA, 2 DPA, 6 DPA, 13 DPA); **i–l** *A. thaliana*


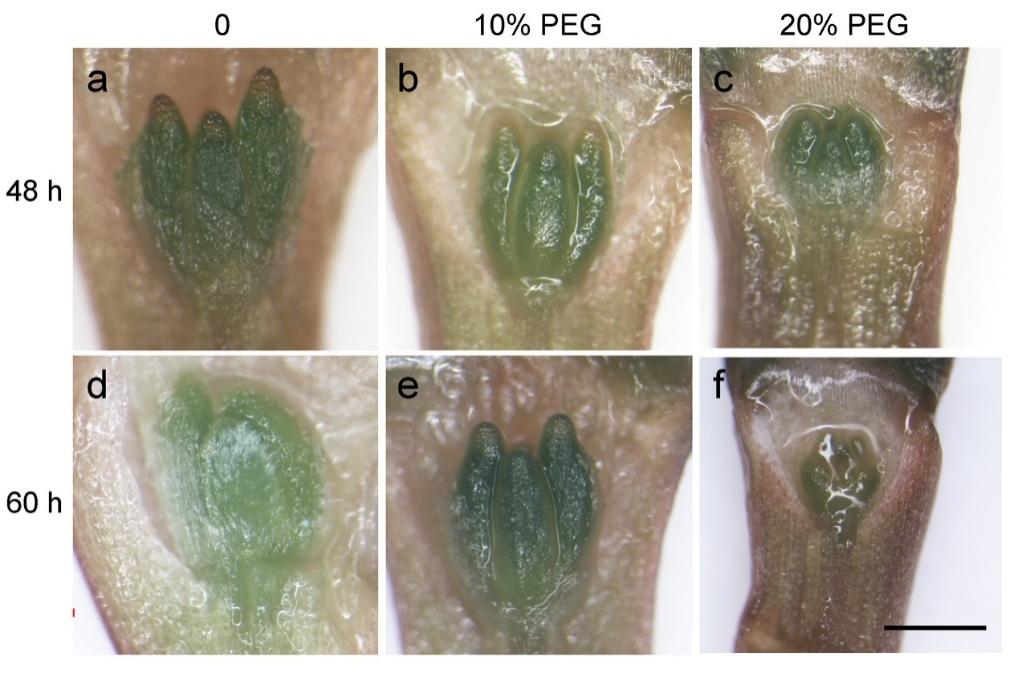


**Fig. S9** The morphology of *S. ferganica* under treatment with different concentrations of PEG 6000. **a–c** 48h d. **d–f** 60 d. Scale bar: **a–f**, 500 μm


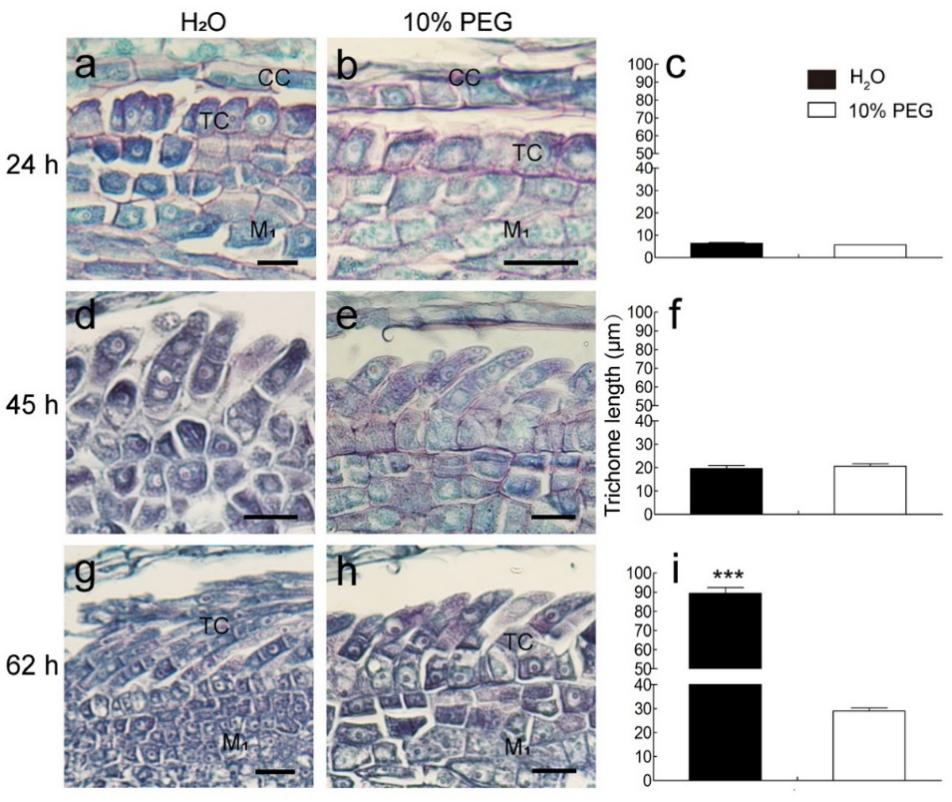


**Fig. S10** Effect of drought stress on trichome development in *S. ferganica*. **a**–**b**, **d**–**e**, **g**–**h** Cytological changes of the trichome cell during true leaf development under PEG treatment. **c**, **f**, **i** Measurements of trichome length corresponding to **a**–**b**, **d**–**e**, **g**–**h**, respectively. **a**–**c** Germination for 24 h; **d**–**f** 45 h; **g**–**i** 62 h; **a**, **d**, **g** H_2_O; **b**, **e**, **h** 10% PEG. Scale bar in **a**, **b**, **d**, **e**, **g**, and **h** is 10 μm; ***: represents a significant difference (*P*<0.05 or 0.01) between different PEG concentrations at the same developmental time. Values are means ± SD of at least seven replicates
